# Supplementary material for: NECTIN4 (PVRL4) as Putative Therapeutic Target for a Specific Subtype of High Grade Serous Ovarian Cancer—An Integrative Multi-Omics Approach
Source: Cancers (Basel). 2019 May 20;11(5):698. doi: 10.3390/cancers11050698 (PMC6562934; doi:10.3390/cancers11050698)
Supplement: Supplementary file 1 [file cancers-11-00698-s001.zip › cancers-491103-suppl-final/cancers-491103-suppl-final.docx]

Supplementary Material

NECTIN4 (PVRL4) as Putative Therapeutic Target for a Specific Subtype of High Grade Serous Ovarian Cancer—An Integrative Multi-Omics Approach

Christine Bekos, Besnik Muqaku, Sabine Dekan, Reinhard Horvat, Stephan Polterauer, Christopher Gerner, Stefanie Aust and Dietmar Pils

**Table S1.** Table of sub-clusters differentially expressed in tumor cells of patients with miliary peritoneal tumor spread compared to patients with non-miliary peritoneal tumor spread (AS, ascites tumor cells; PM, solid tumor tissues). Static plots (with regulation information, red, up in miliary, green, down in miliary, each with gene names labelled if significant and darker colored and always name-labelled, if hub genes) and interactive network-representations with genes (nodes) linked to GeneCards (http://www.genecards.org/) of the first three Clusters are linked and can be shown by a mouse click on the links (Folder: “*Tables S1 and S2*”).

**Table S2.** Genes of sub-cluster *c1_143* with information of different expression between miliary and non-miliary solid (PM) or ascites derived floating (AS) tumor cells and usage in the gene signature for survival validation as shown in Figure 2H (Folder: “*Tables S1 and S2*”).

**Table S3.** Univariate and multiple Cox regression analysis for progression free survival in 90 patients with late stage (FIGO III/IV) high grade serous ovarian cancer.

| **Progression Free Survival** | | | | | | |  |
| --- | --- | --- | --- | --- | --- | --- | --- |
| **Cox regression analysees** | **Univariate ^1^** | | | **Multiple ^2^** | | |  |
|  | **HR** | **CI_95_** | ***p*** | **HR** | **CI_95_** | ***p*** | |
| **Age**  (decades) | **1.41** | **1.13–1.77** | **0.003** | **1.92** | **1.11–1.76** | **0.004** | |
| **FIGO stage**  (IV vs. III) | **1.95** | **1.16–3.30** | **0.012** | **1.74** | **1.02–2.98** | **0.043** | |
| Histological grade  (G3 vs. G2) | 1.00 | 0.56–1.76 | 0.989 | 0.86 | 0.47–1.55 | 0.606 | |
| **Residual tumor**  (R1 vs. R0) | **2.57** | **1.58–4.21** | **<0.001** | **2.27** | **1.36–3.77** | **0.002** | |
| **Nectin 4**  (>50% vs. ≤50%)^3^ | **2.60** | **1.33–5.08** | **0.005** | 1.92 | 0.94–3.95 | 0.074 | |

^1^ Univariate Cox-regression; ^2^ Multiple Cox-regression analysis; HR, Hazard Ratio; CI_95_, 95% confidence interval. ^3^ The optimal cutoff was assessed by non-linear modeling of the Nectin 4 impact on OS by fractional polynomials Cox regression estimation correcting for known clinicopathologic factors age, FIGO stage, grade, and residual tumor mass after debulking surgery. In Figure 3A the corrected relative hazard against the percentage of Nectin 4 positive tumor cells is shown indicating a negative impact on OS in tumors with >50%, and therefore this cutoff, >50% versus ≤50%, was used for outcome analyses. Bold, statistically significant.

**Table S4.** Univariate and multiple Cox regression analysis for overall survival in 90 patients with late stage (FIGO III/IV) high grade serous ovarian cancer including pre-operative CA 125 levels and chemotherapy mode.

| **Overall Survival** | | | | | | |  |
| --- | --- | --- | --- | --- | --- | --- | --- |
| Cox regression analyses | **Univariate ^1^** | | | **Multiple ^2^** | | |  |
|  | **HR** | **CI_95_** | ***p*** | **HR** | **CI_95_** | ***p*** | |
| **Age**  (decades) | See Table 3 | | | **2.27** | **1.55–3.34** | **<0.001** | |
| FIGO stage  (IV vs. III) |  |  |  | 1.38 | 0.60–3.17 | 0.452 | |
| Histological grade  (G3 vs. G2) |  |  |  | 0.58 | 0.24–1.41 | 0.227 | |
| Residual tumor  (R1 vs. R0) |  |  |  | 1.36 | 0.64–2.89 | 0.431 | |
| CA 125 (log_10_) | 0.92 | 0.57–1.51 | 0.750 | 0.76 | 0.46–1.28 | 0.302 | |
| **Chemotherapy**  (neoadj. Vs. adj.) | **2.46** | **1.01–6.04** | **0.049** | 1.16 | 0.38–3.58 | 0.790 | |
| **Nectin 4**  (>50% vs. ≤50%) ^3^ | **3.03** | **1.37–6.68** | **0.006** | **4.64** | **1.52–14.13** | **0.007** | |

^1^Univariate Cox-regression; ^2^Multiple Cox-regression analysis; HR, Hazard Ratio; CI_95_, 95% confidence interval. ^3^The optimal cutoff was assessed by non-linear modeling of the Nectin 4 impact on OS by fractional polynomials Cox regression estimation correcting for known clinicopathologic factors age, FIGO stage, grade, and residual tumor mass after debulking surgery. In Figure 3A the corrected relative hazard against the percentage of Nectin 4 positive tumor cells is shown indicating a negative impact on OS in tumors with >50%, and therefore this cutoff, >50% versus ≤50%, was used for outcome analyses. Bold, statistically significant.

**Table S5.** Tables of analytes (cf. Table 4) with their associations with the protein Nectin 4 score (0, negative; 1, ≤50%; 2, >50%). logFC, log_2_ fold change; False Discovery Rate (FDR) = adj.P.Val = pGFdr *(File: “Table S5.xlsx”)*.


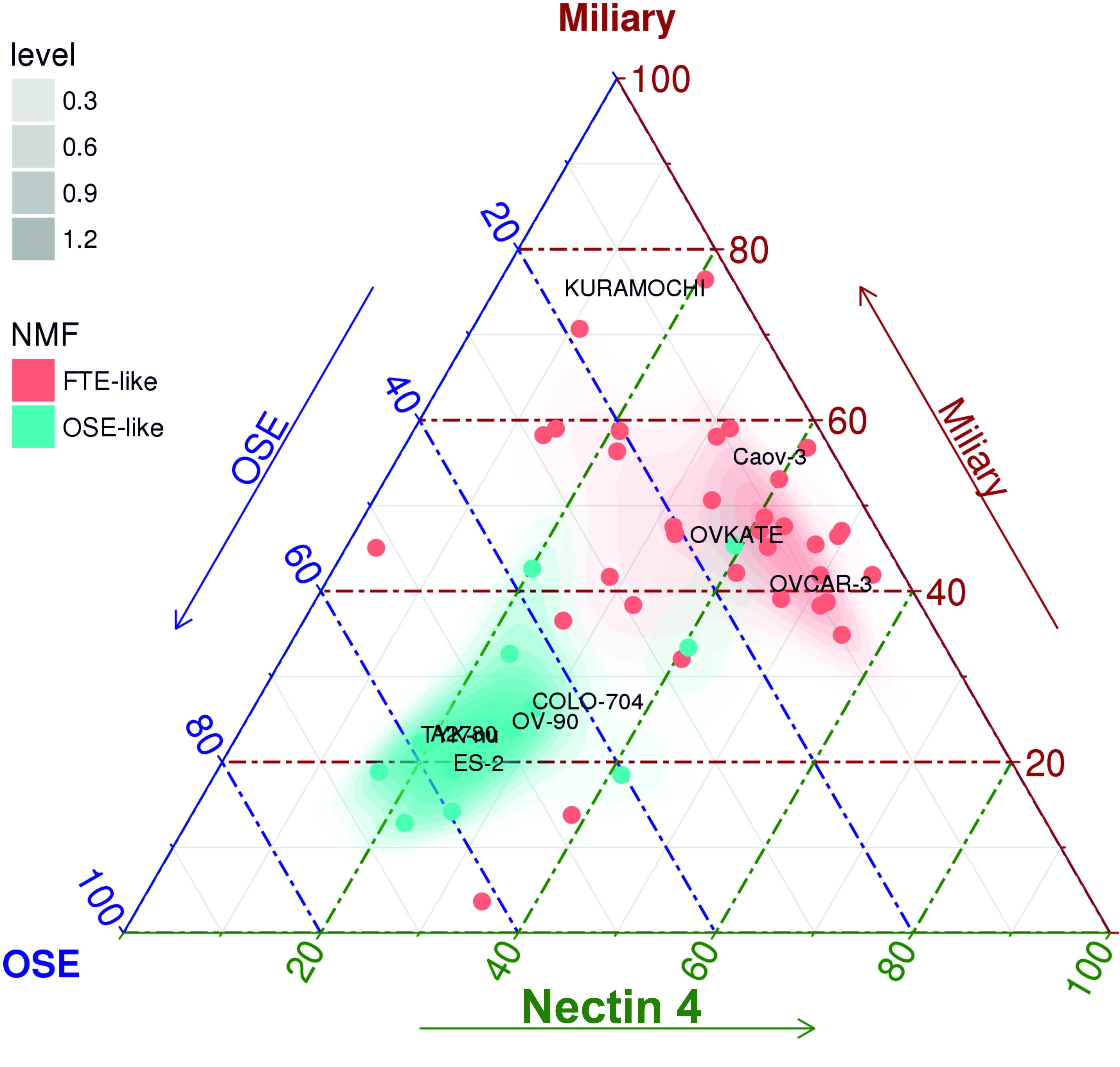


**Figure S1.** 48 ovarian cancer cell lines characterized according the miliary-versus-non-miliary and the origin gene signatures (right and left edges, respectively) and Nectin 4 gene expression. Each four cell lines most probably derived of high grade serous tumors of both origins (tube or ovary) and spread types (miliary or non-miliary) are labeled by their names and all cell lines are colored according a non-negative matrix factorization procedure (NMF; red for FTE(tube)-like and green for OSE(ovarian)-like). For further details see Material and Methods.


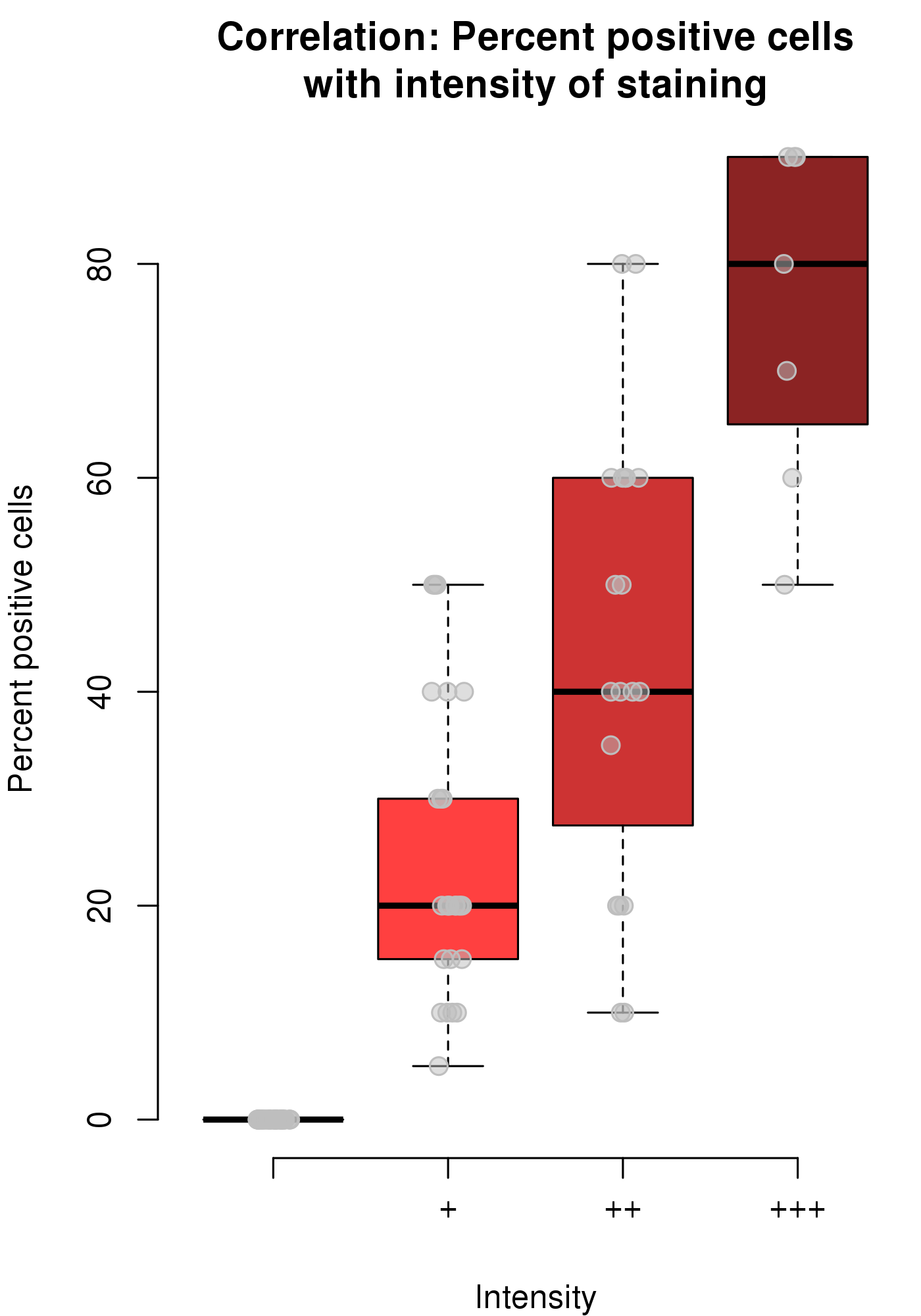


**Figure S2.** Correlation of staining intensities and percentages of Nectin 4 positive cells on tumor tissues of 90 HGSOC patients.


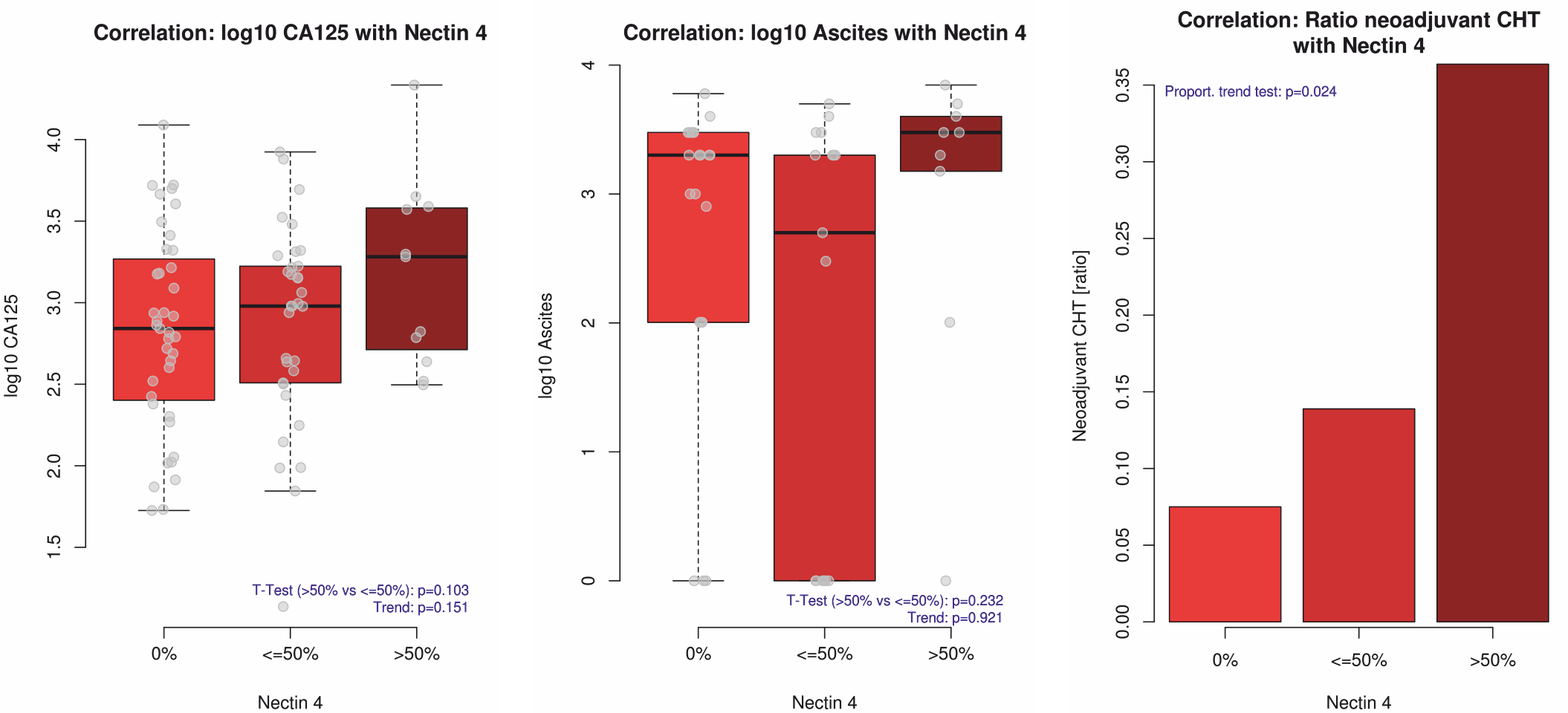


**Figure S3.** Correlation of CA 125 (log_10_ U mL^-1^), amount of ascites (log_10_ _mL_), and chemotherapy mode (ratio neoadjuvant to all (neoadj.+adjuvant)) with the Nectin 4 score (*X*-axis). *p*-values are not corrected for multiple testing.

**
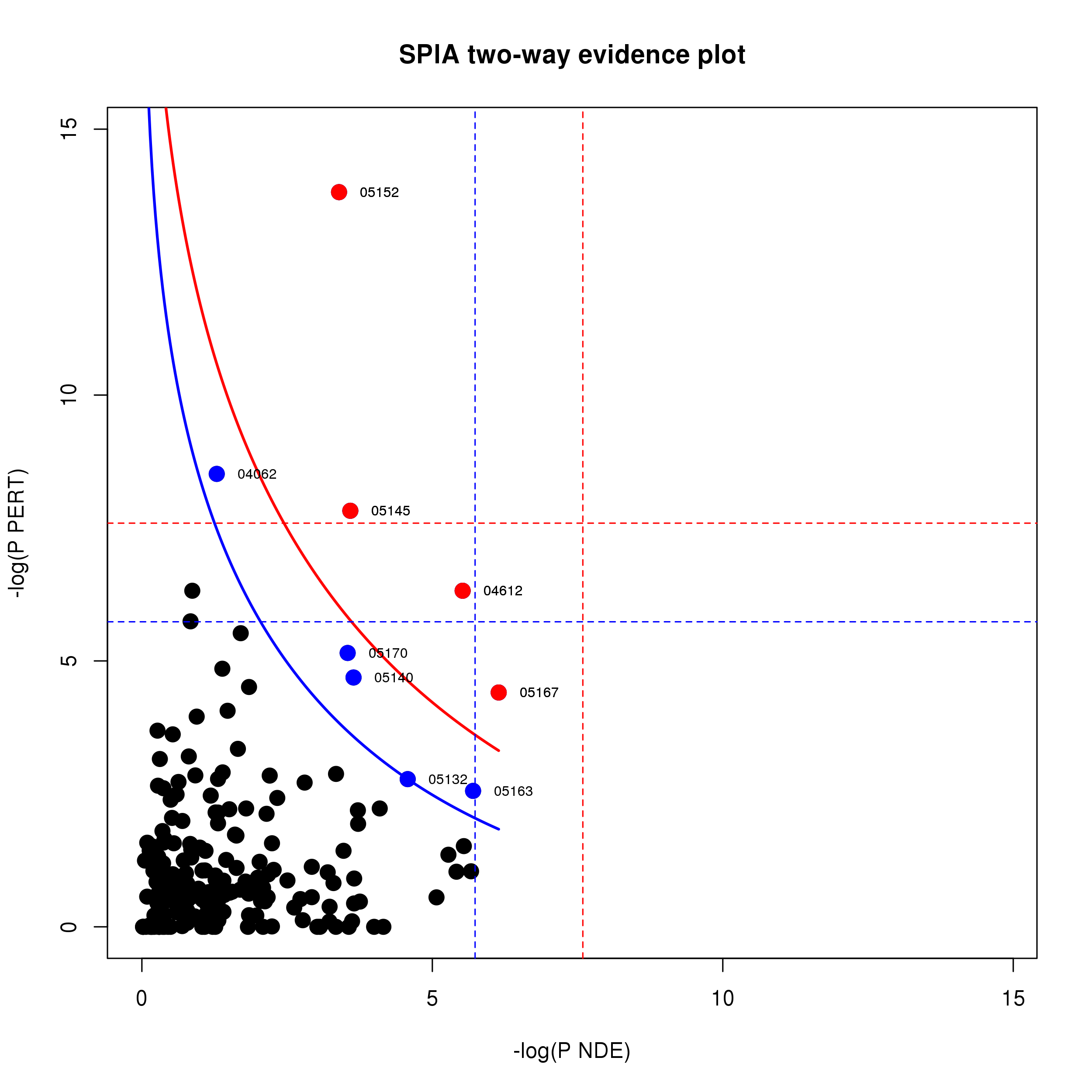
 Figure S4.** SPIA evidence plot of significantly deregulated KEGG pathways. Each pathway is represented by one dot. The pathways at the right of the blue oblique line are significant after a FDR correction (<10%) of the global *p*-values, pG, obtained by combining the pPERT (corresponding to the probability of obtaining the observed total accumulation (tA) or more extreme on the given pathway just by chance) and pNDE (corresponding to the probability of obtaining at least the observed number of genes (NDE)) using “norminv” method. The red line is not used.


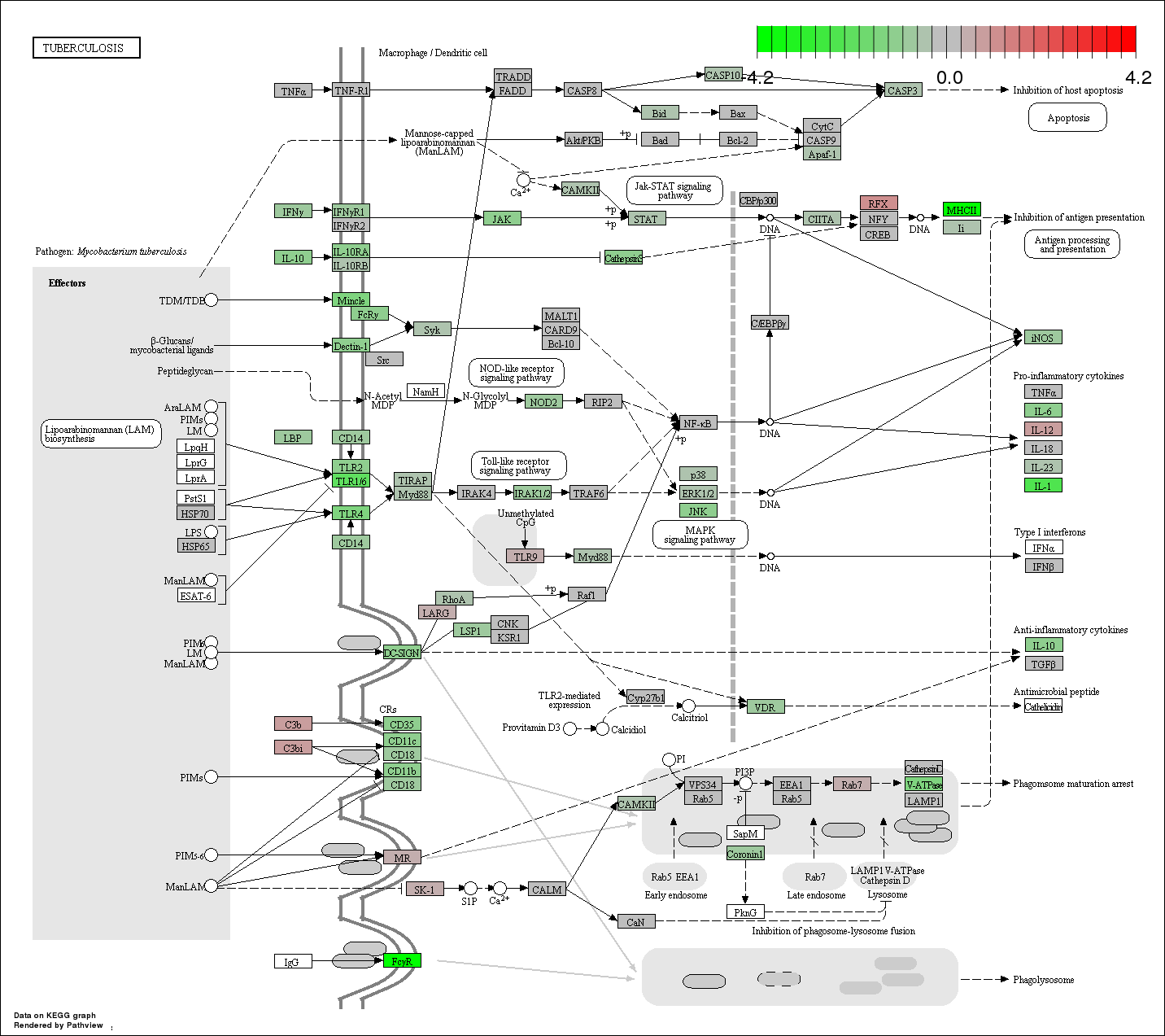


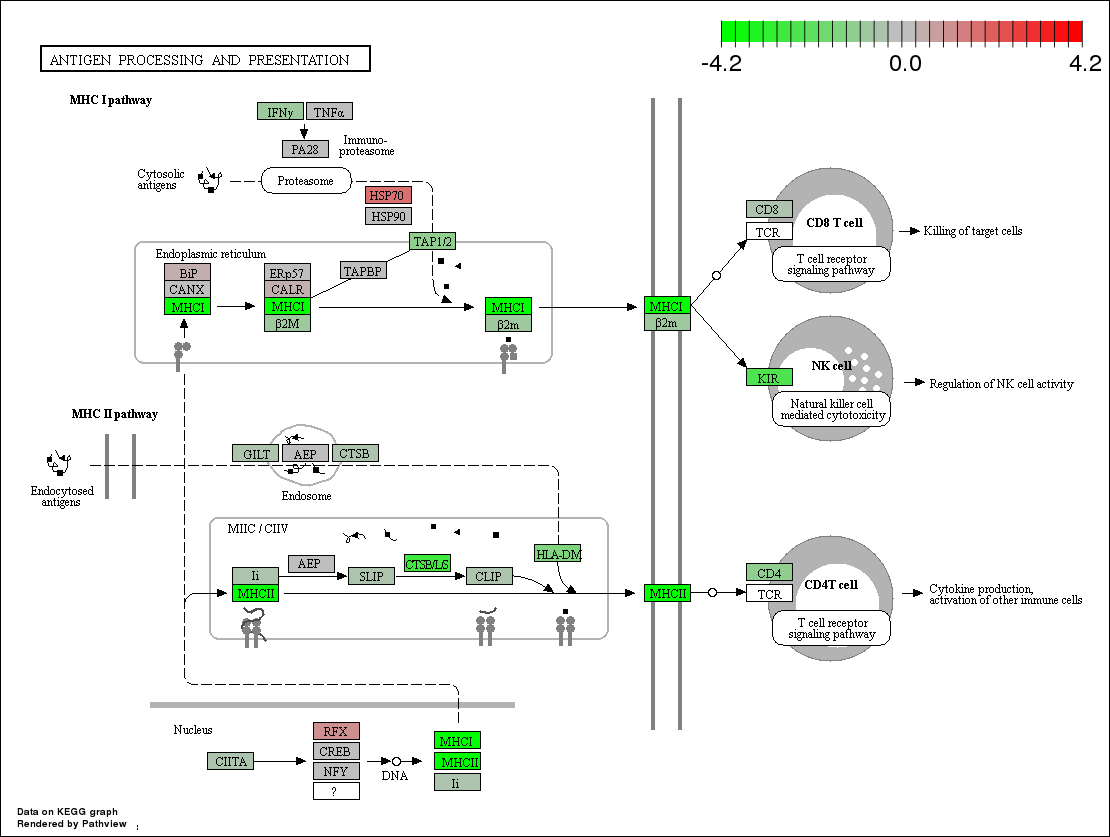


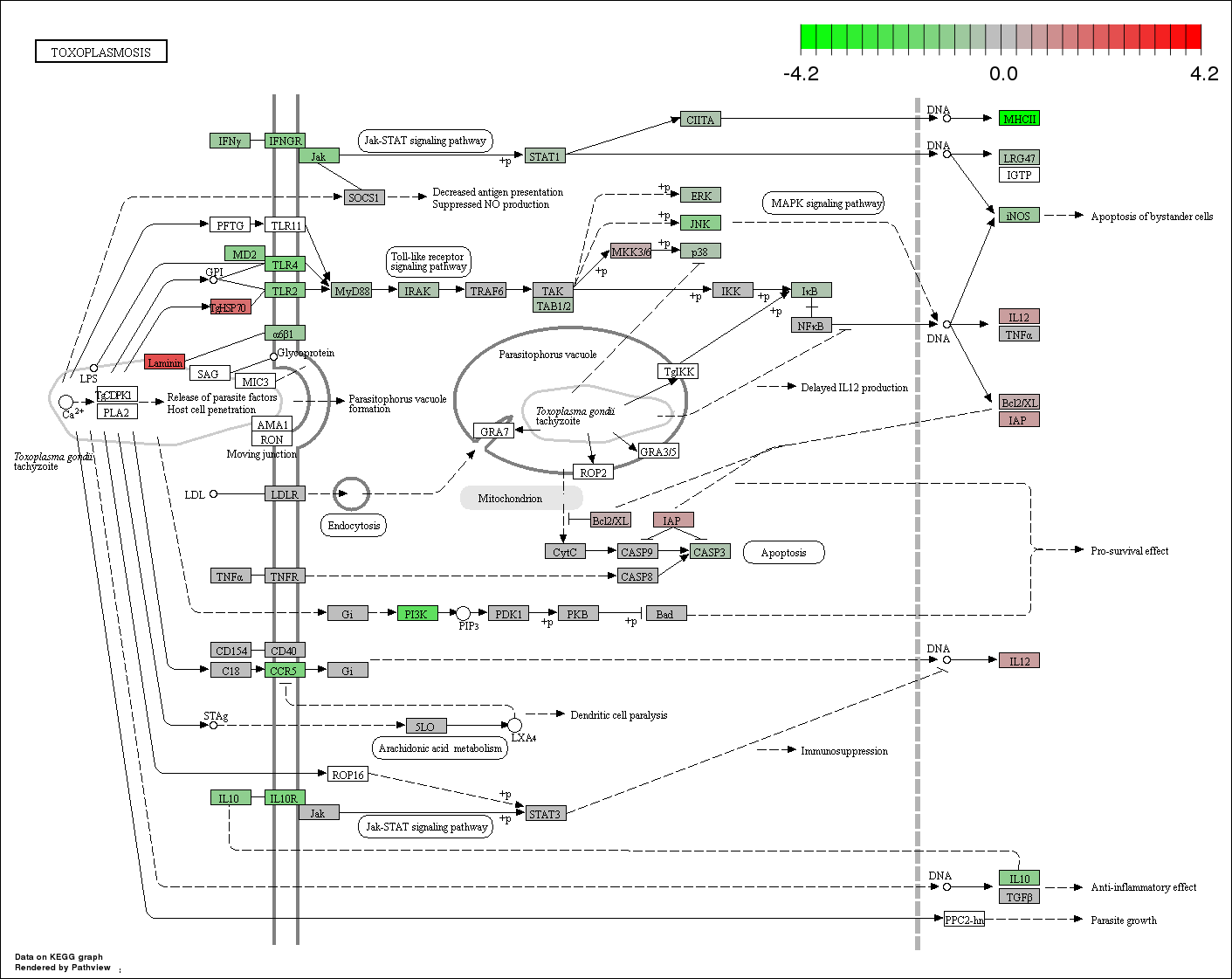


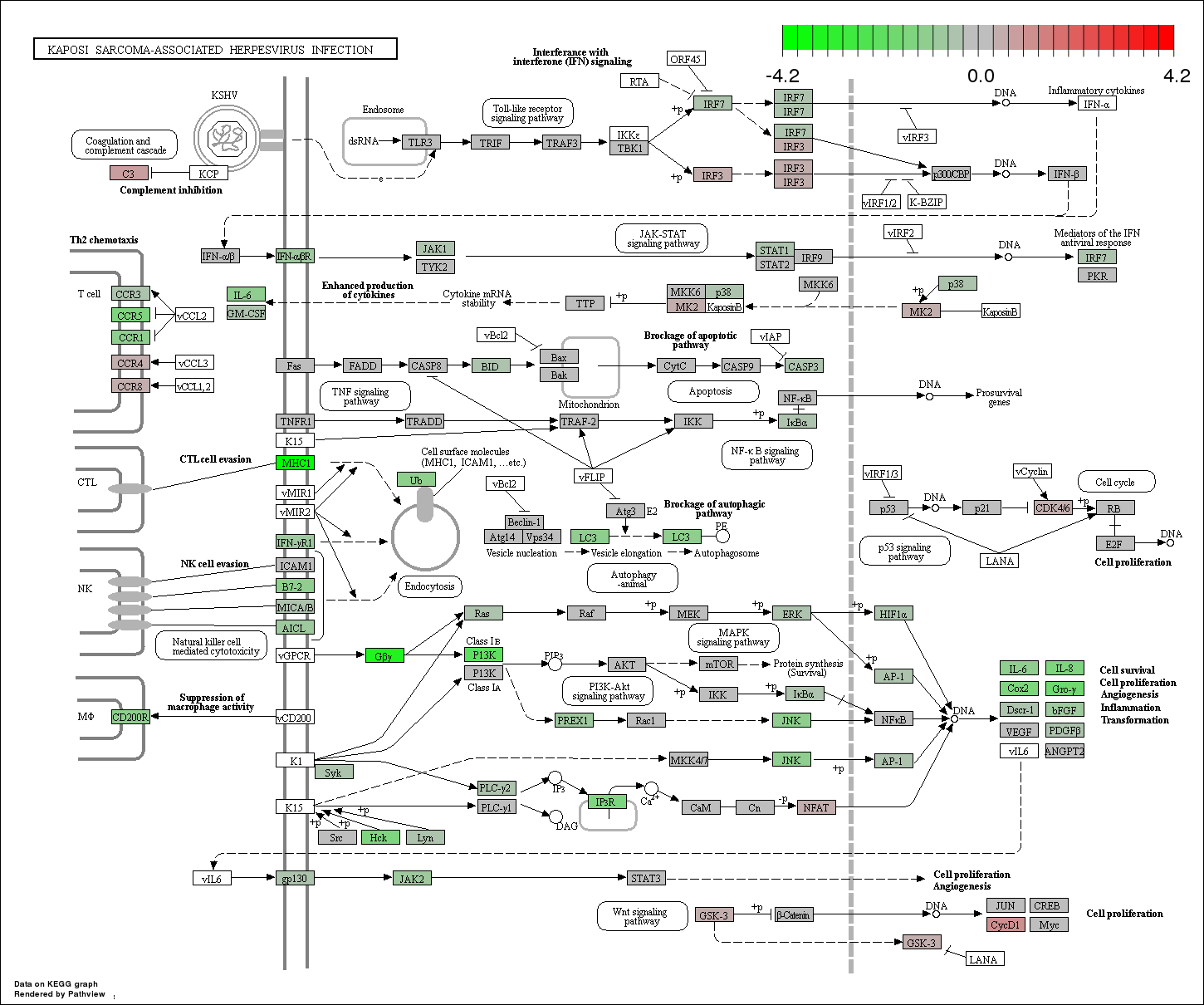


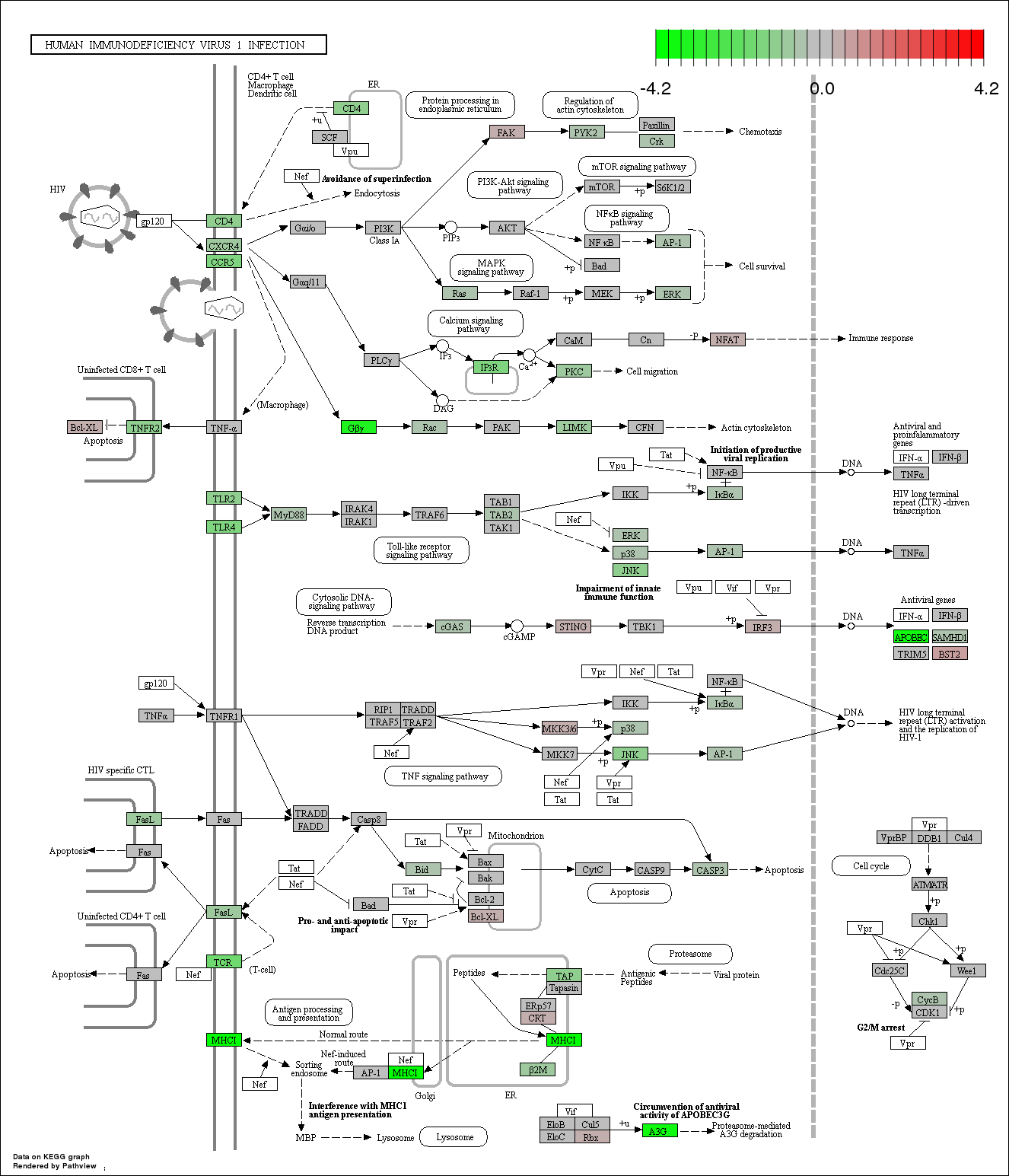


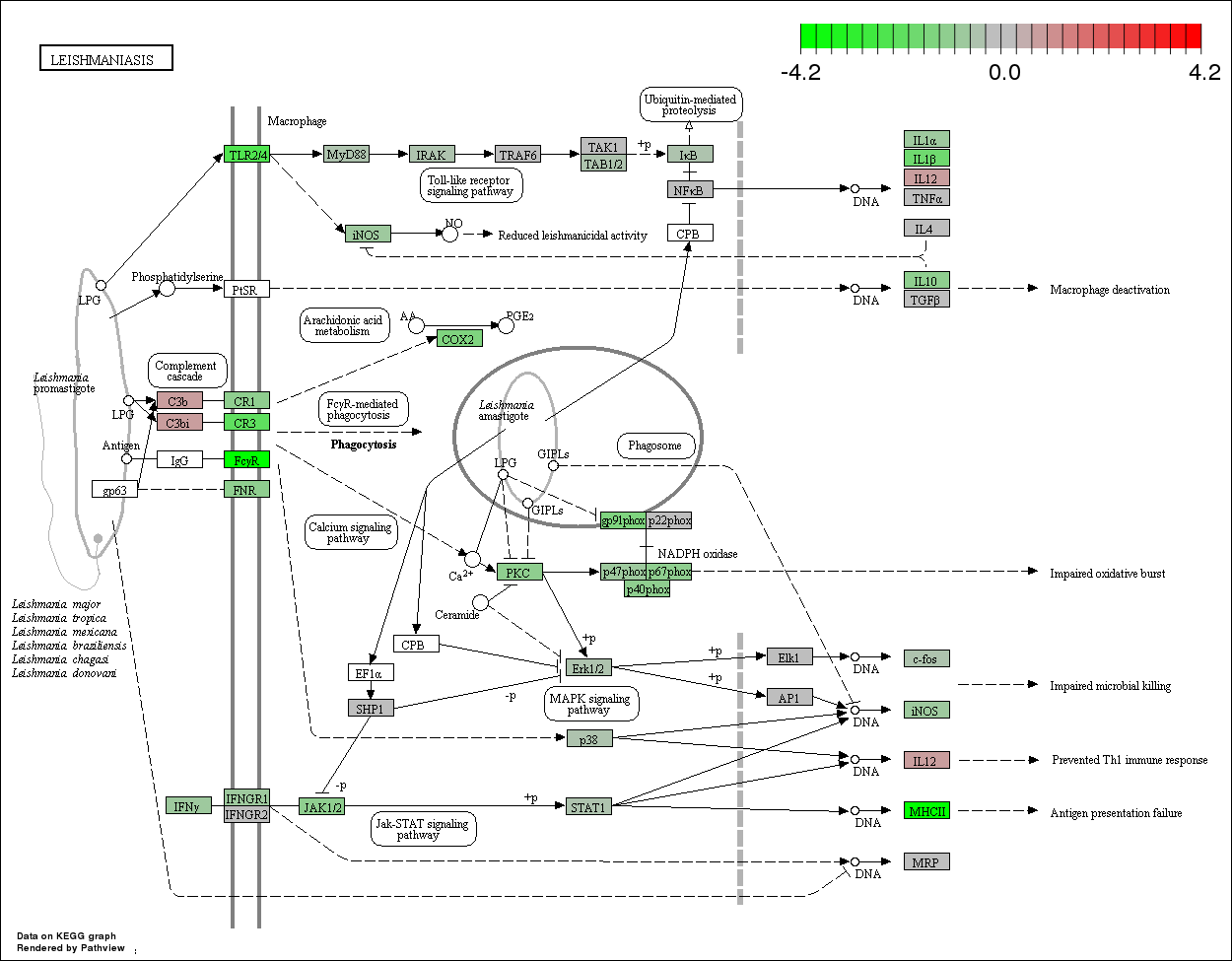


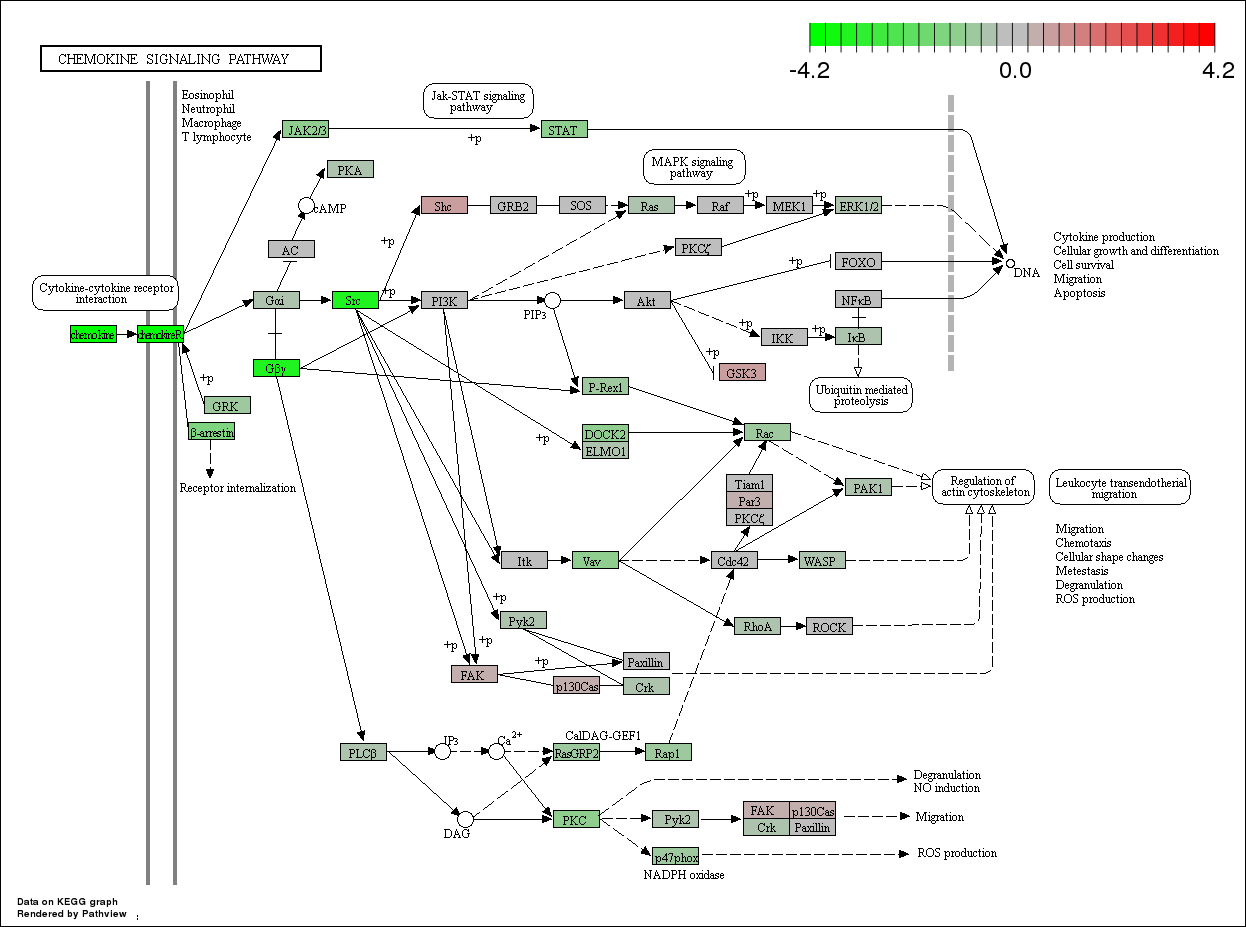


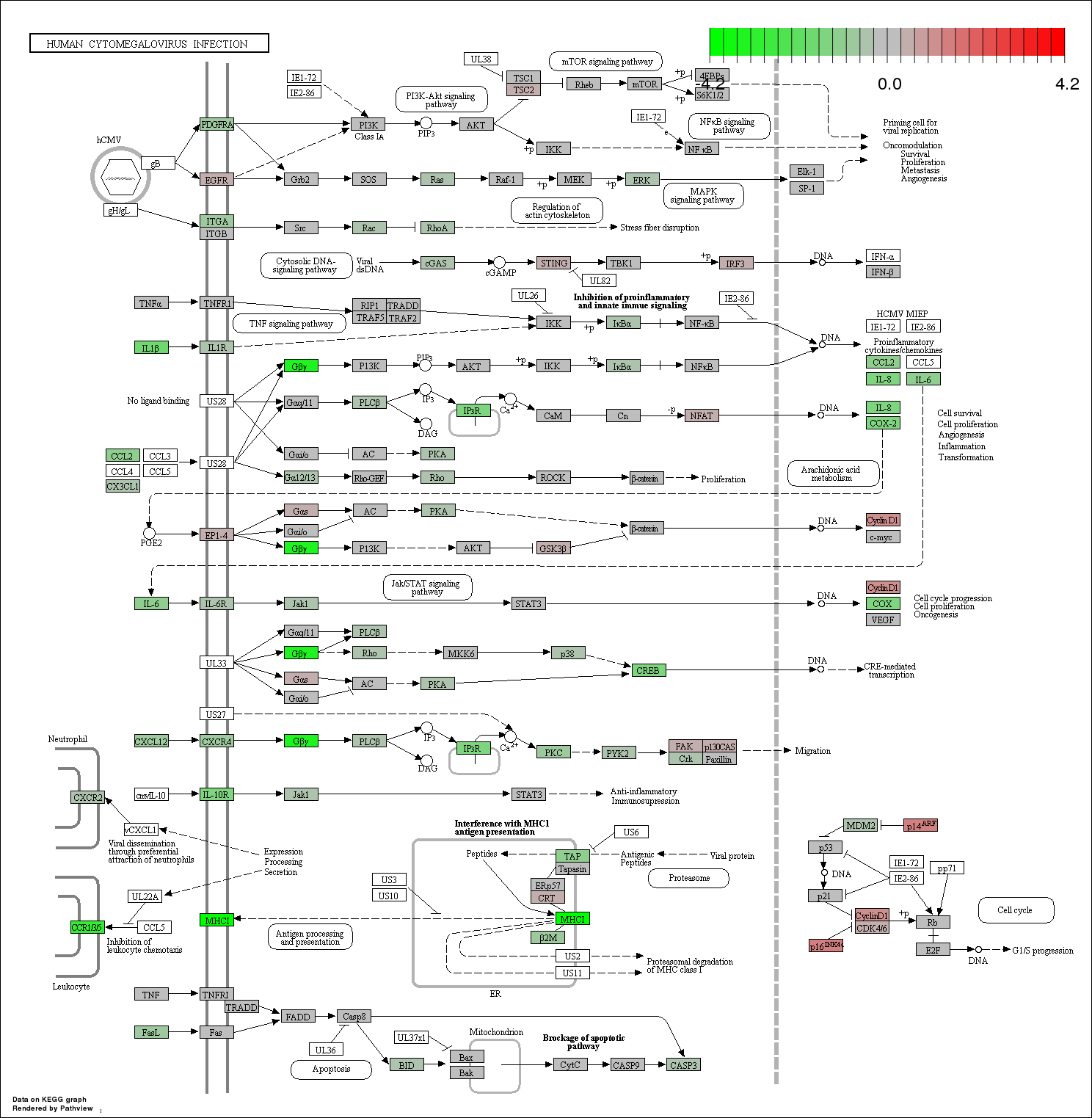


**Figures S5–12.** Significantly downregulated KEGG pathways. Colors represent log_2_ fold changes according correlations to the Nectin 4 score. White nodes were not represented in the RNA-sequencing data, either because they were not (reliably) expressed or could not be mapped.

**
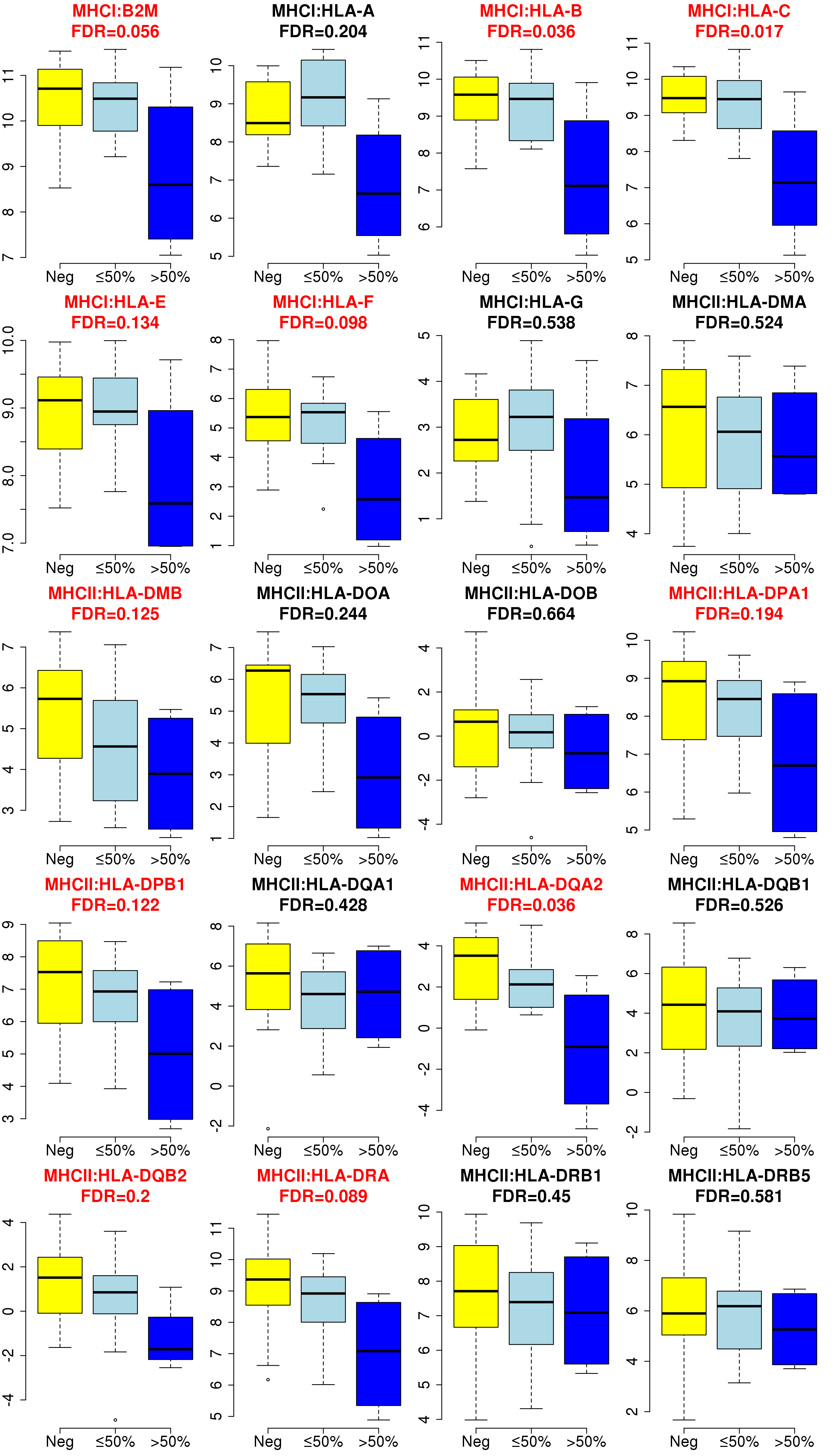
**

**Figure S13.** Coding HLA genes (major histocompatibility complexes I (MHCI) and II (MHCII)) correlated to the Nectin 4 score. Headers in red indicate significance at the FDR 20% level.

**
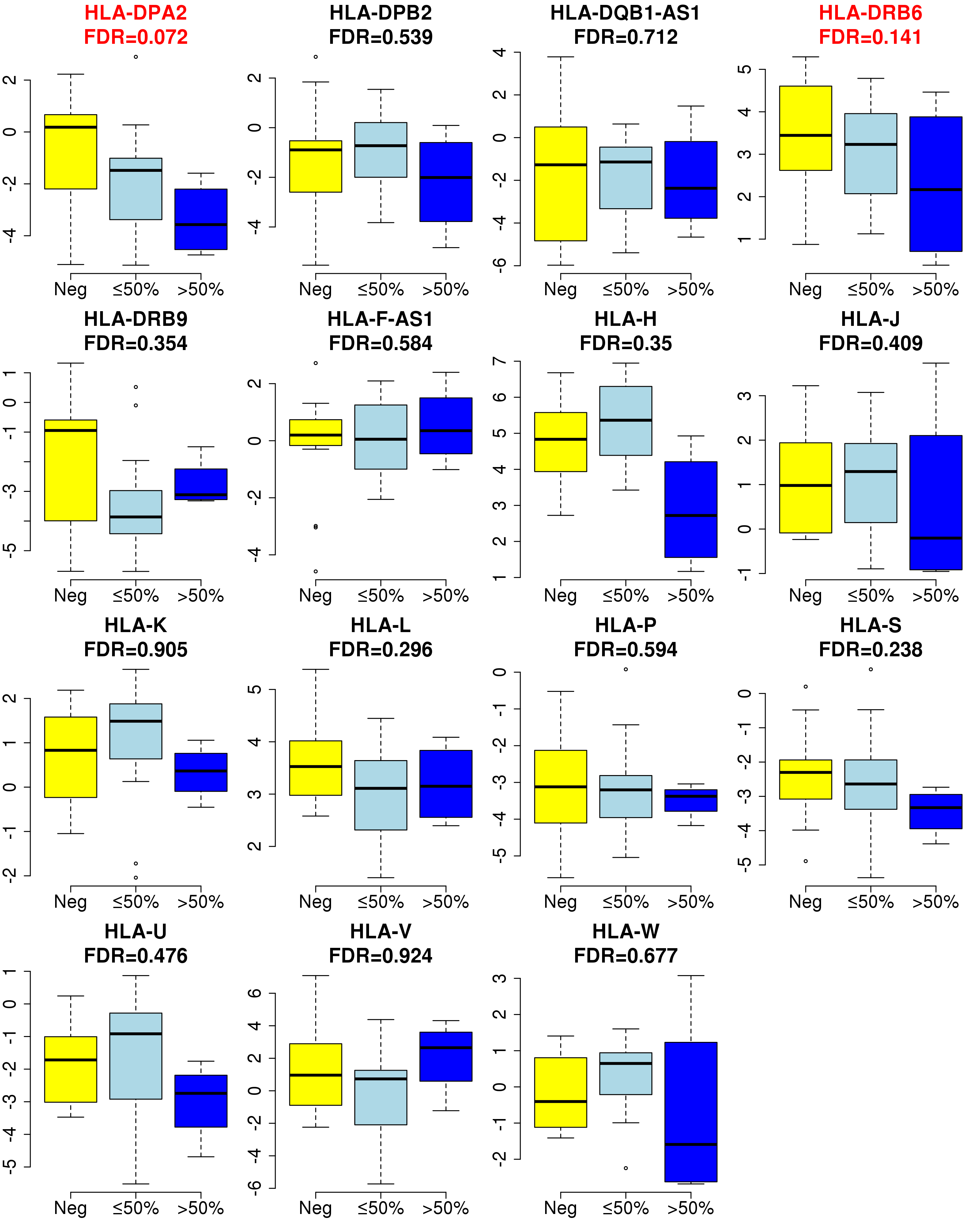
**

**Figure S14.** Non-coding (pseudo- or antisens-) HLA genes correlated to the Nectin 4 score. Headers in red indicate significance at the FDR 20% level.

**
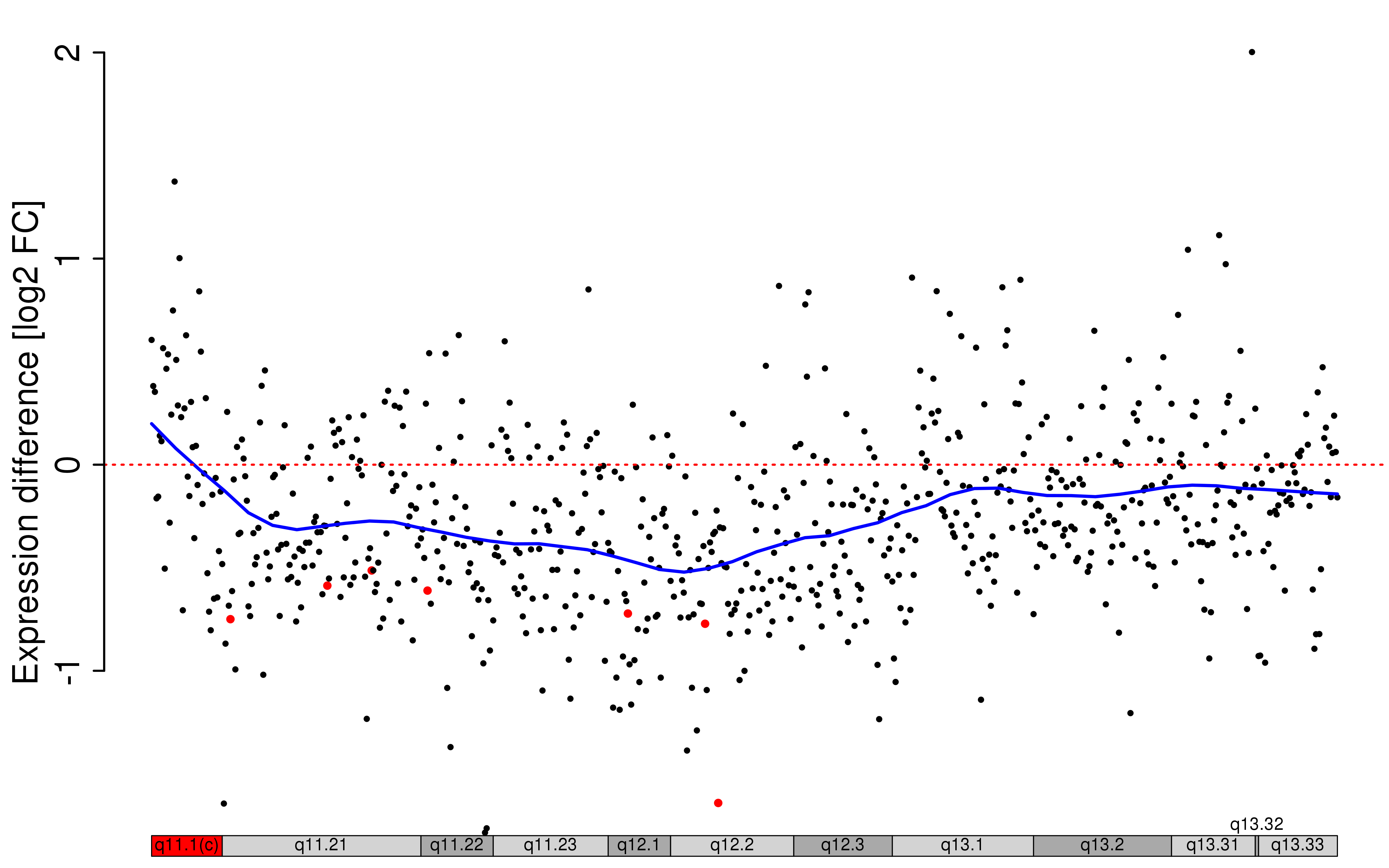
**

**Figure S15.** Log_2_ fold-changes associated to the Nectin 4 score of all expressed genes on chromosome 22. Genes were ordered according their relative positions on chromosome 22. The length of the indicated chromosomal bands correspond not to the physical length of the bands on the chromosome. Red labeled are the seven genes from the high-scoring sub-networks, significantly associated to the Nectin 4 score (cf. Figure S31).

**
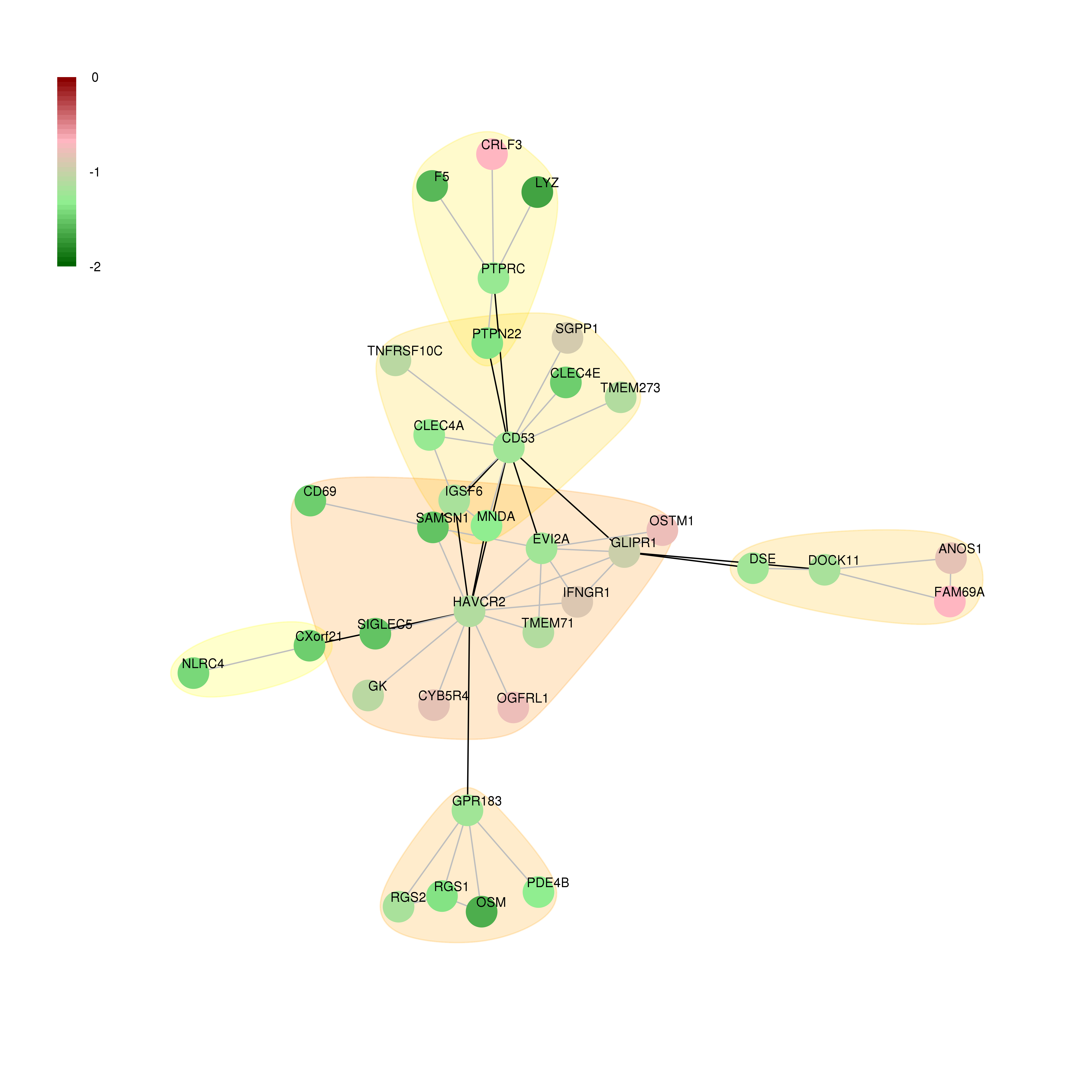
**

**Figure S16.** Nectin 4 score associated high-scoring sub-network of the TCGA co-association gene expression network TCGAnet. Color: log_2_ fold changes according Nectin 4 score associations; cave: all log_2_ fold change values are negative, therefore the color bar is from −2 to 0.


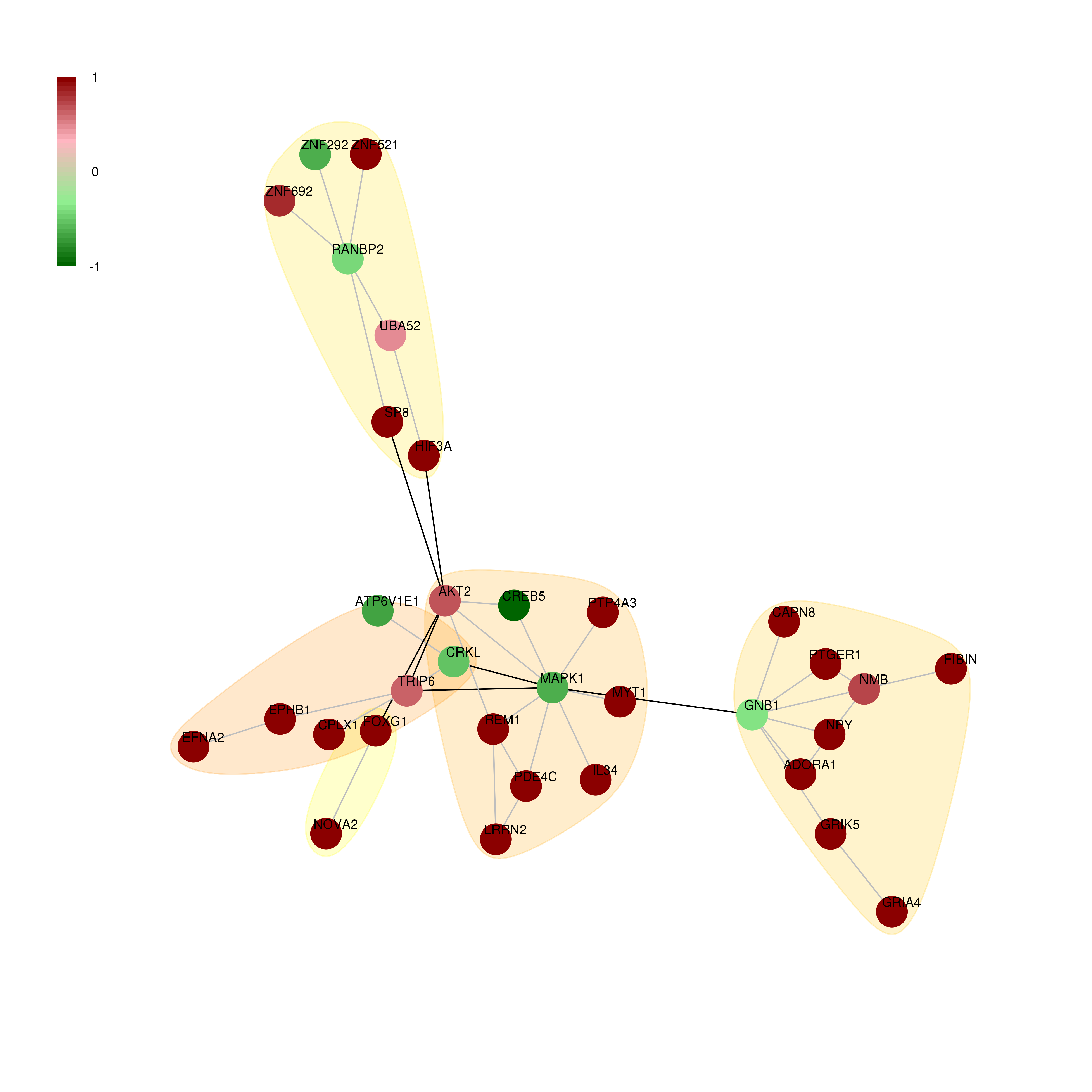


**Figure S17.** Nectin 4 score associated high-scoring sub-network of the functional protein-protein interaction network STRING v10. Color: log_2_ fold changes according Nectin 4 score associations.


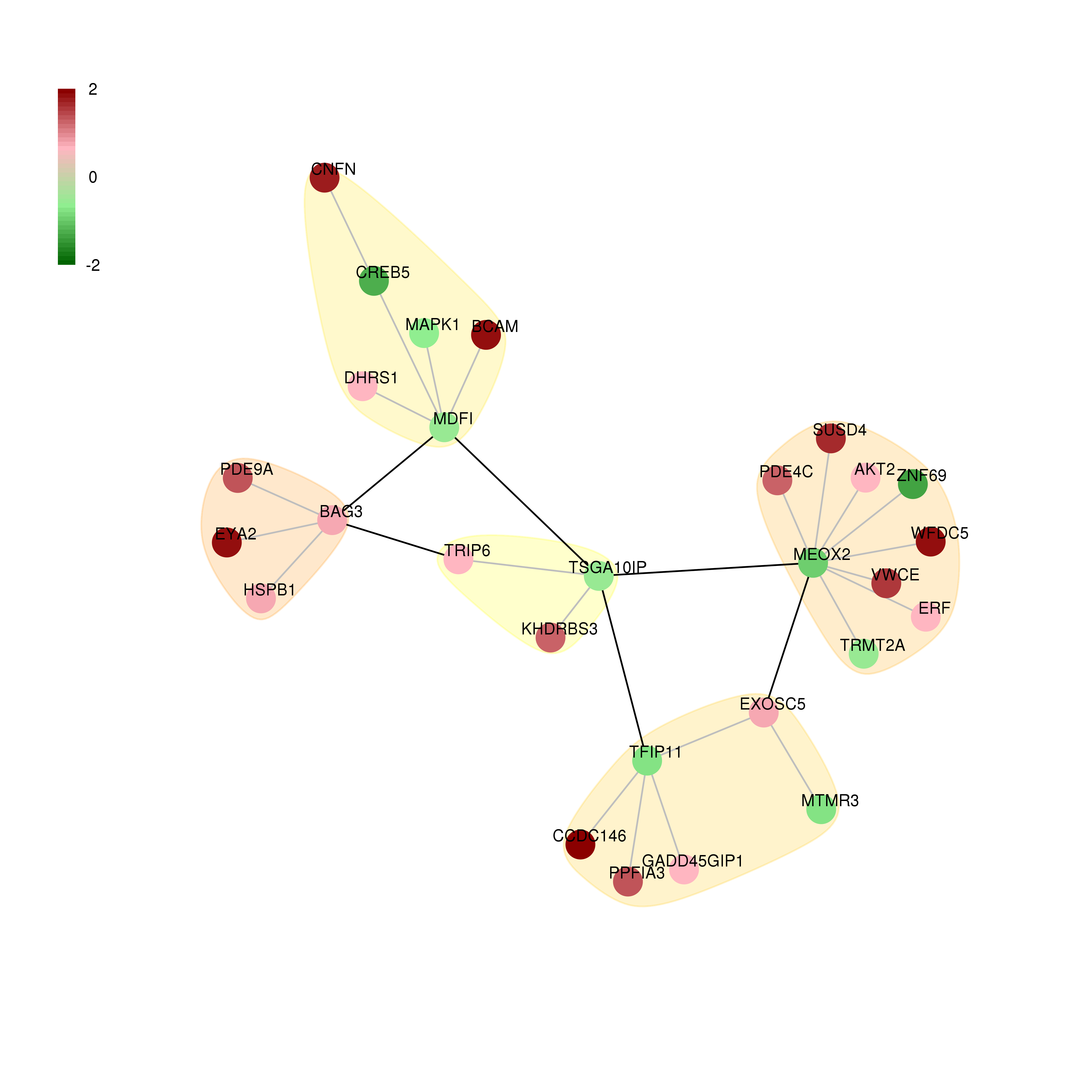


**Figure S18.** Nectin 4 score associated high-scoring sub-network of the experimentally verified protein-protein interaction network (CCSB Human Interactome database, HI-III, preliminary release 2.5). Color: log_2_ fold changes according Nectin 4 score associations.


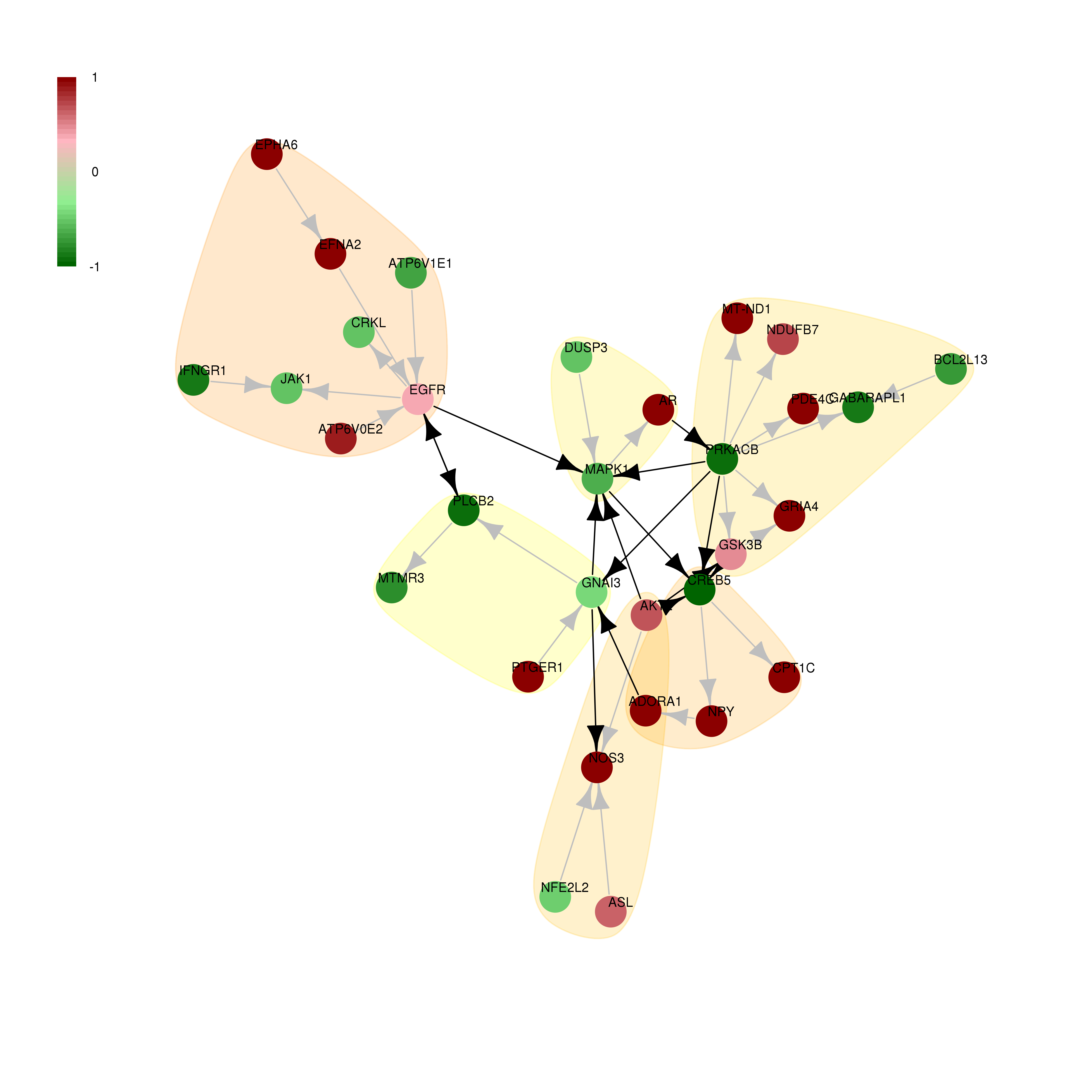


**Figure S19.** Nectin 4 score associated high-scoring sub-network of the KEGG pathway super-network (all KEGG pathways connected). Color: log_2_ fold changes according Nectin 4 score associations.

**

**

**20: c1_180.**

**

**

**21: c1_315.**

**

**

**22: c1_335.**

**

**

**23: c1_358.**

**

**

**24: c1_363.**

**

**

**25: c1_376.**

**

**

**26: c1_496.**

**

**

**27: c1_539.**

**

**

**28: c1_747.**

**

**

**29: c1_760.**

**
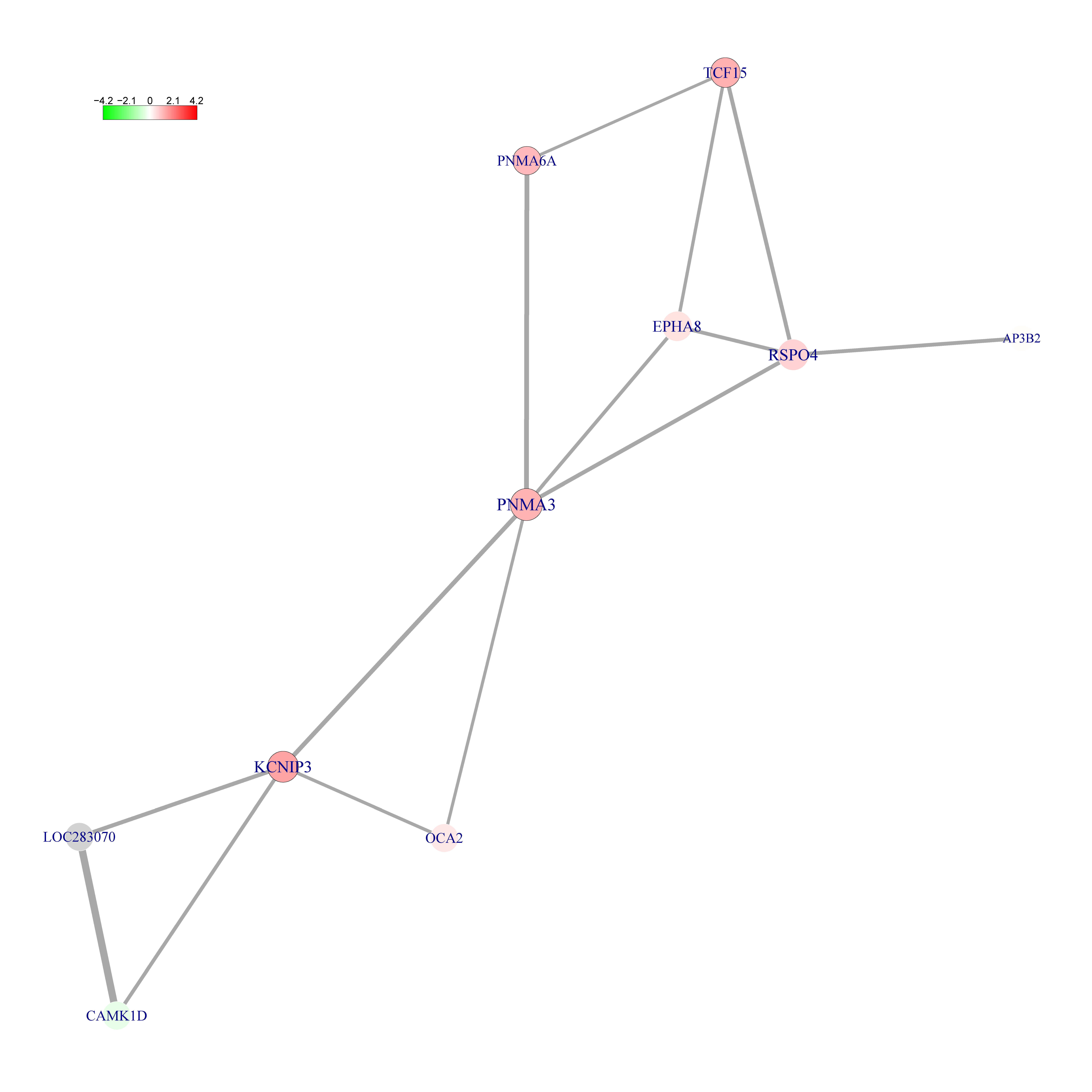
**

**30: c1_782.**

**Figures S20–30.** TCGA co-association clusters significantly associated with the Nectin 4 score. Only clusters with less than 50 nodes are shown, and these clusters are together with cluster c1_143 (cf. Figure 3) and the high-scoring networks (TCGAnet, STRING, PPI, and KEGG; cf. Supp. Figures 16–19) the basis of the integrative network shown in Figure 4. Color: log_2_ fold changes according Nectin 4 score associations. If the association is significant (FDR < 5%), the node is framed by a black line.

**
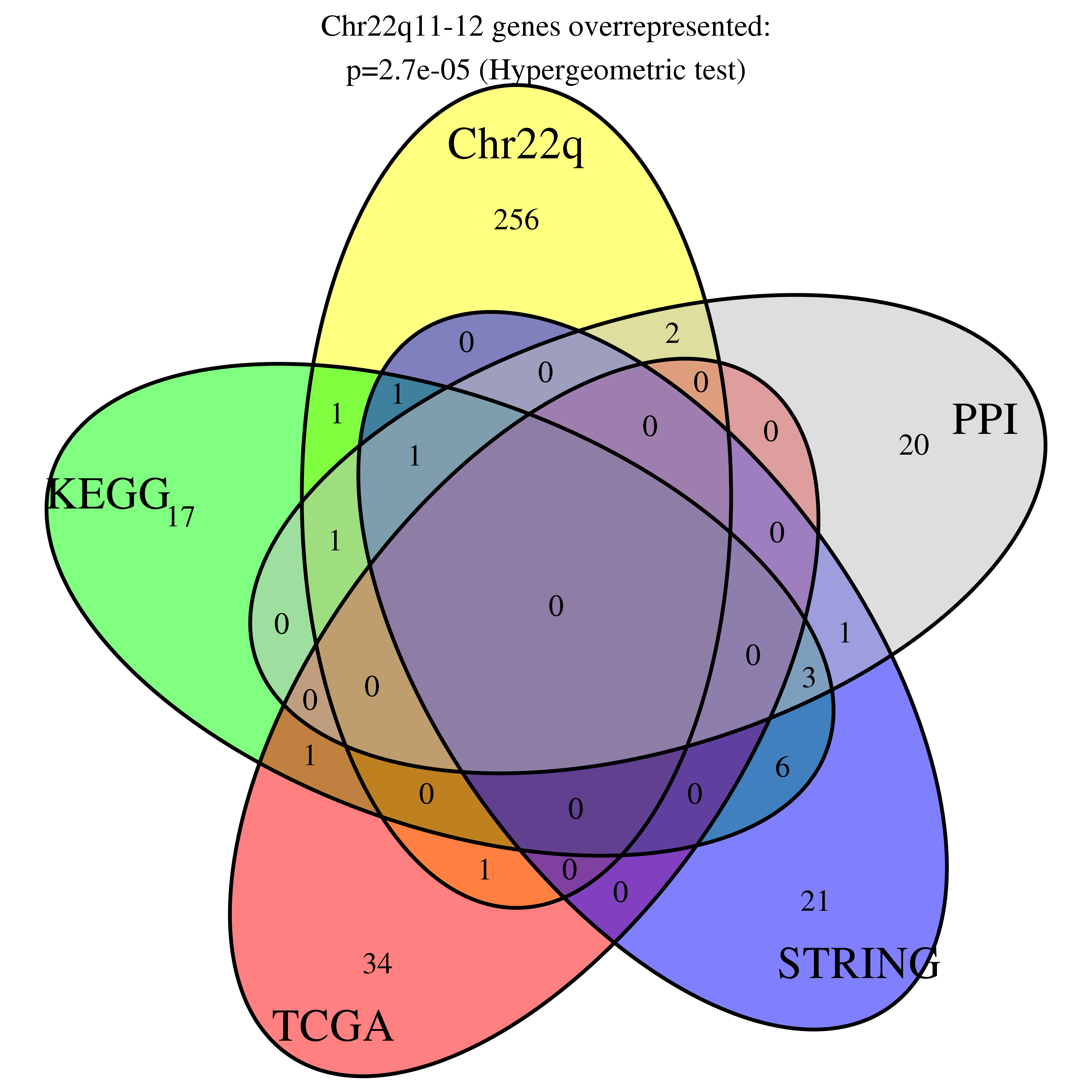
**

**Figure S31.** Overlap of 110 genes from the high-scoring networks (cf. Figures S16–19) with genes from chromosomal bands 22q11-q12 (cf. Figure S15).


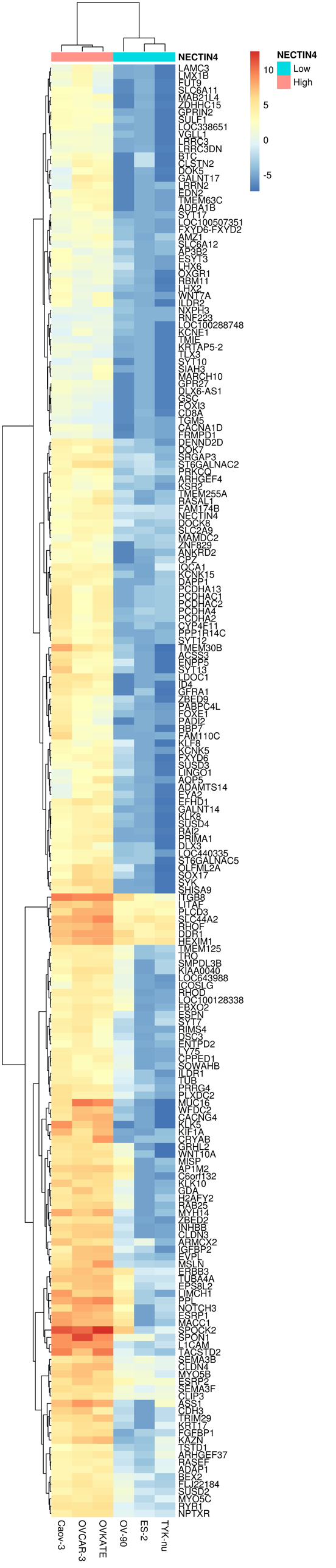


**Figure S32.** Heatmap of differentially expressed genes between three NECTIN4 high (Caov-3, OVCAR-3, and OVKATE) and three NECTIN4 low (OV-90, ES-2, and TYK-nu) expressing HGSOC cell lines (cutoff: FDR < 10% and log2 fold change > 2).


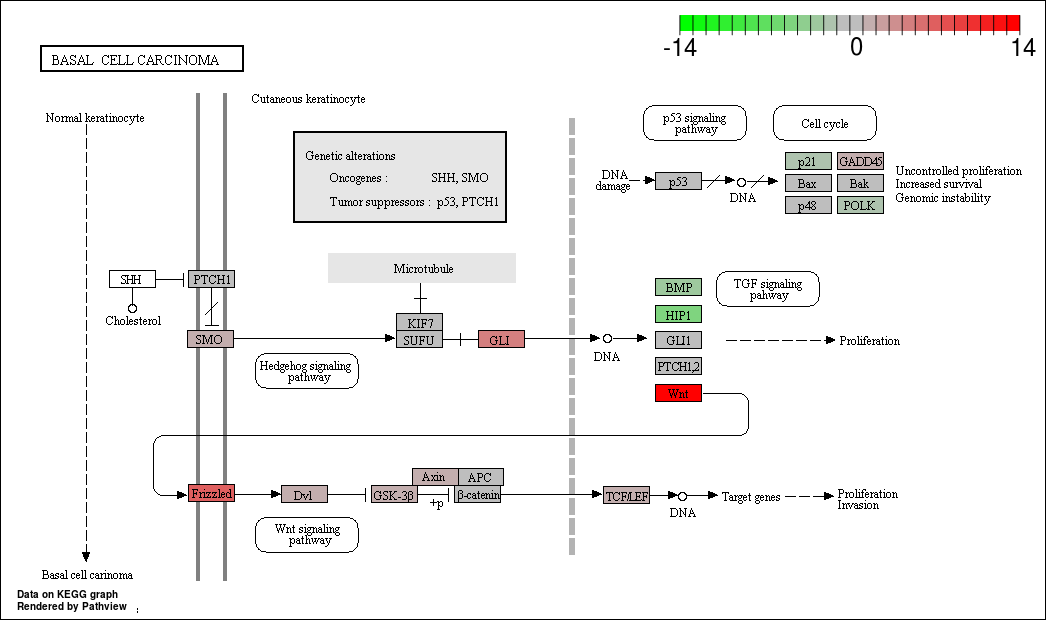


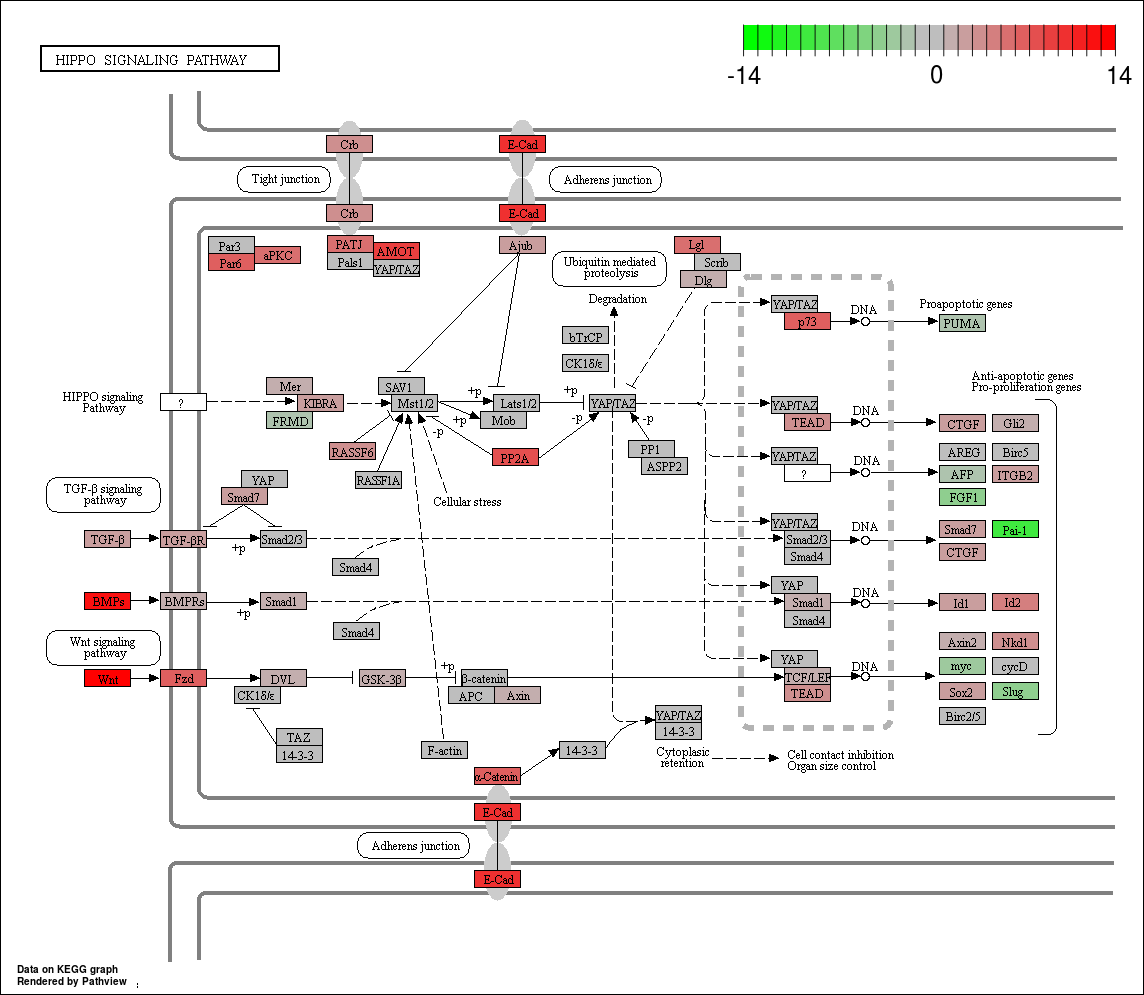


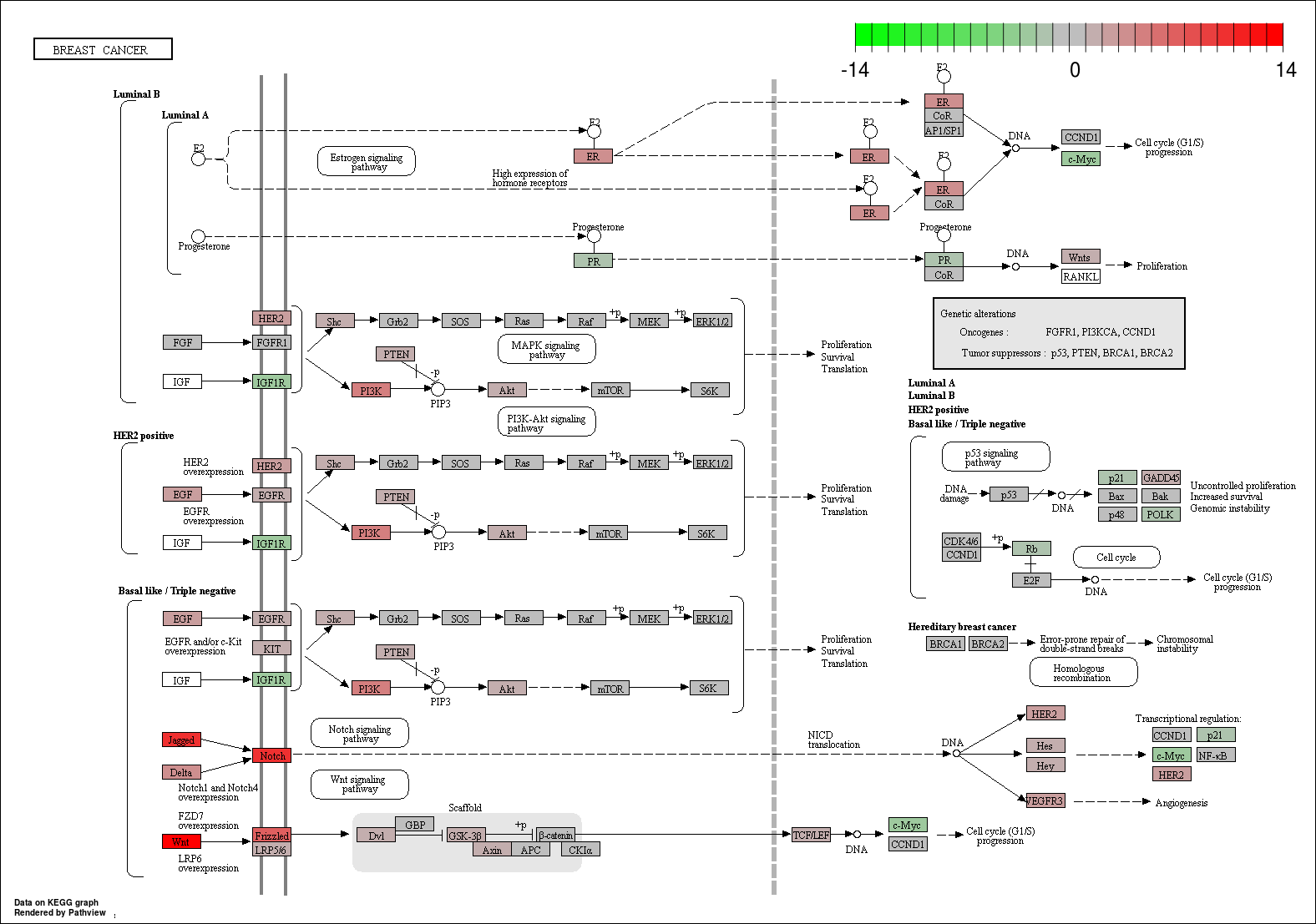


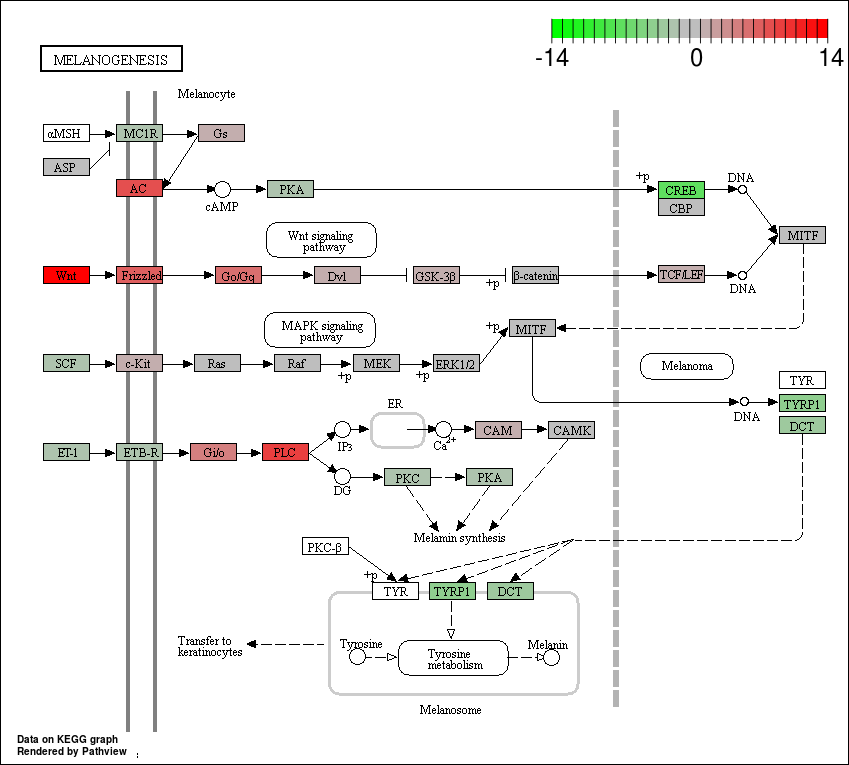


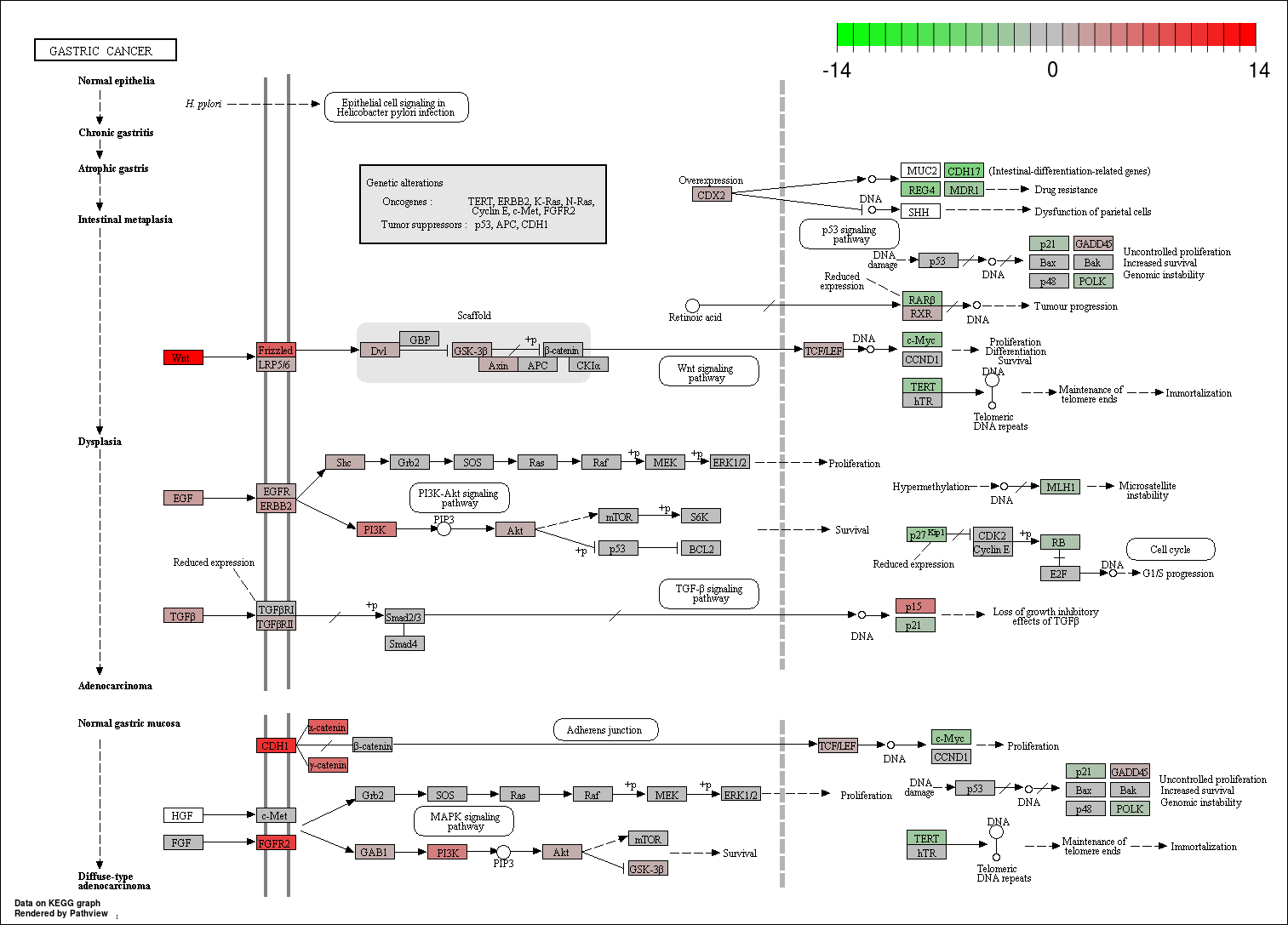


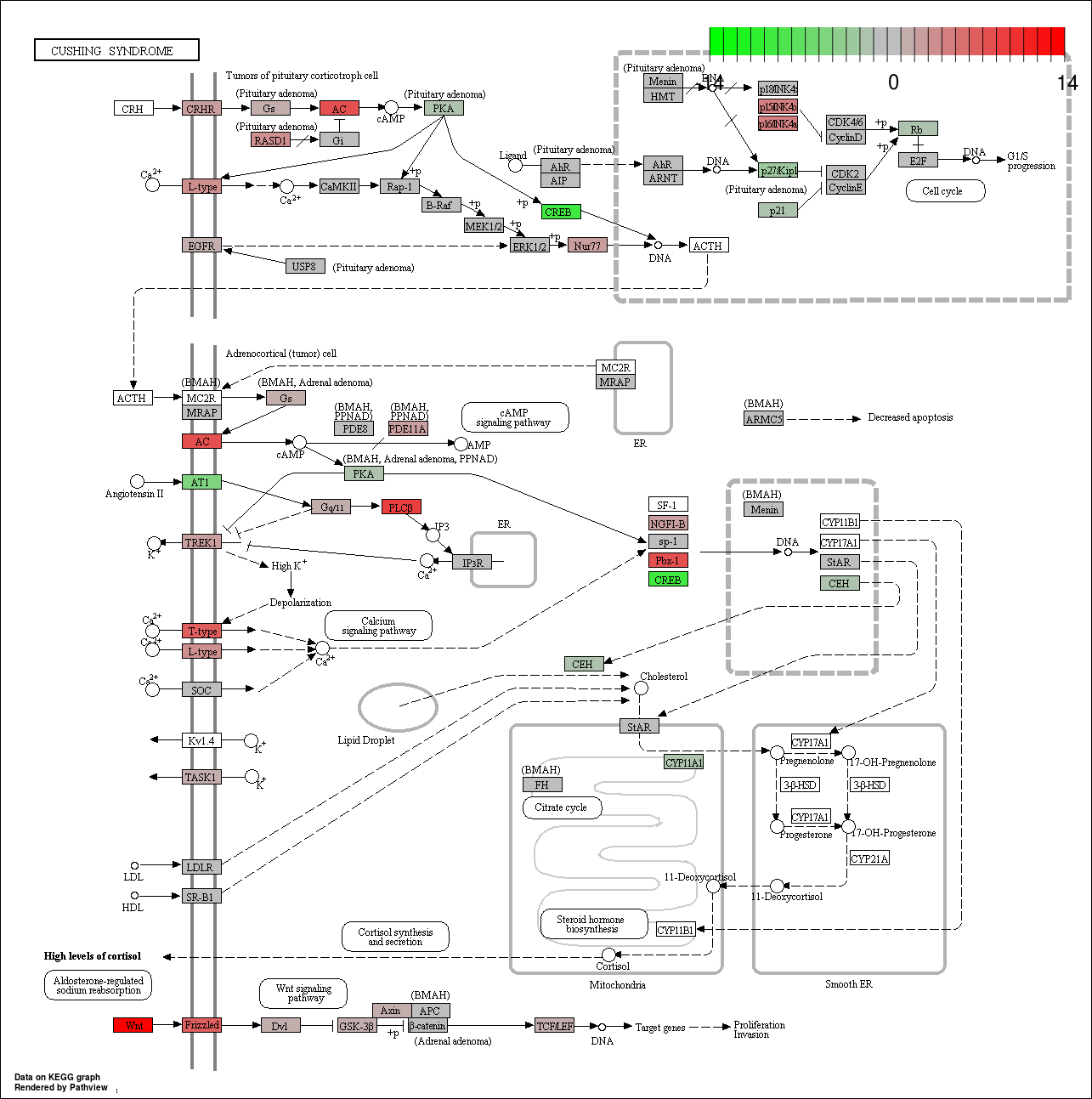


**Figures S33–38.** Significantly (20% FDR) activated KEGG pathways. Colors represent log_2_ fold changes according correlations to NECTIN4 expression. White nodes were not represented in the RNA-sequencing data, either because they were not (reliably) expressed or could not be mapped.

**Figure S39.** Zoomable version of Figure 5 as own pdf file. For legend see legend of Figure 5 (*File: “Figure S39.pdf”*).

| 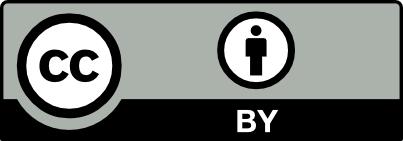 | © 2019 by the authors. Licensee MDPI, Basel, Switzerland. This article is an open access article distributed under the terms and conditions of the Creative Commons Attribution (CC BY) license (http://creativecommons.org/licenses/by/4.0/). |
| --- | --- |
