# Supplementary material for: NECTIN4 (PVRL4) as Putative Therapeutic Target for a Specific Subtype of High Grade Serous Ovarian Cancer—An Integrative Multi-Omics Approach
Source: Cancers (Basel). 2019 May 20;11(5):698. doi: 10.3390/cancers11050698 (PMC6562934; doi:10.3390/cancers11050698)
Supplement: Supplementary file 1 [file c1_143_AS_Up__42_graph.pdf › cancers-491103-suppl-final/Tables S1 and S2/c1_143_AS_Up__42_graph.pdf]

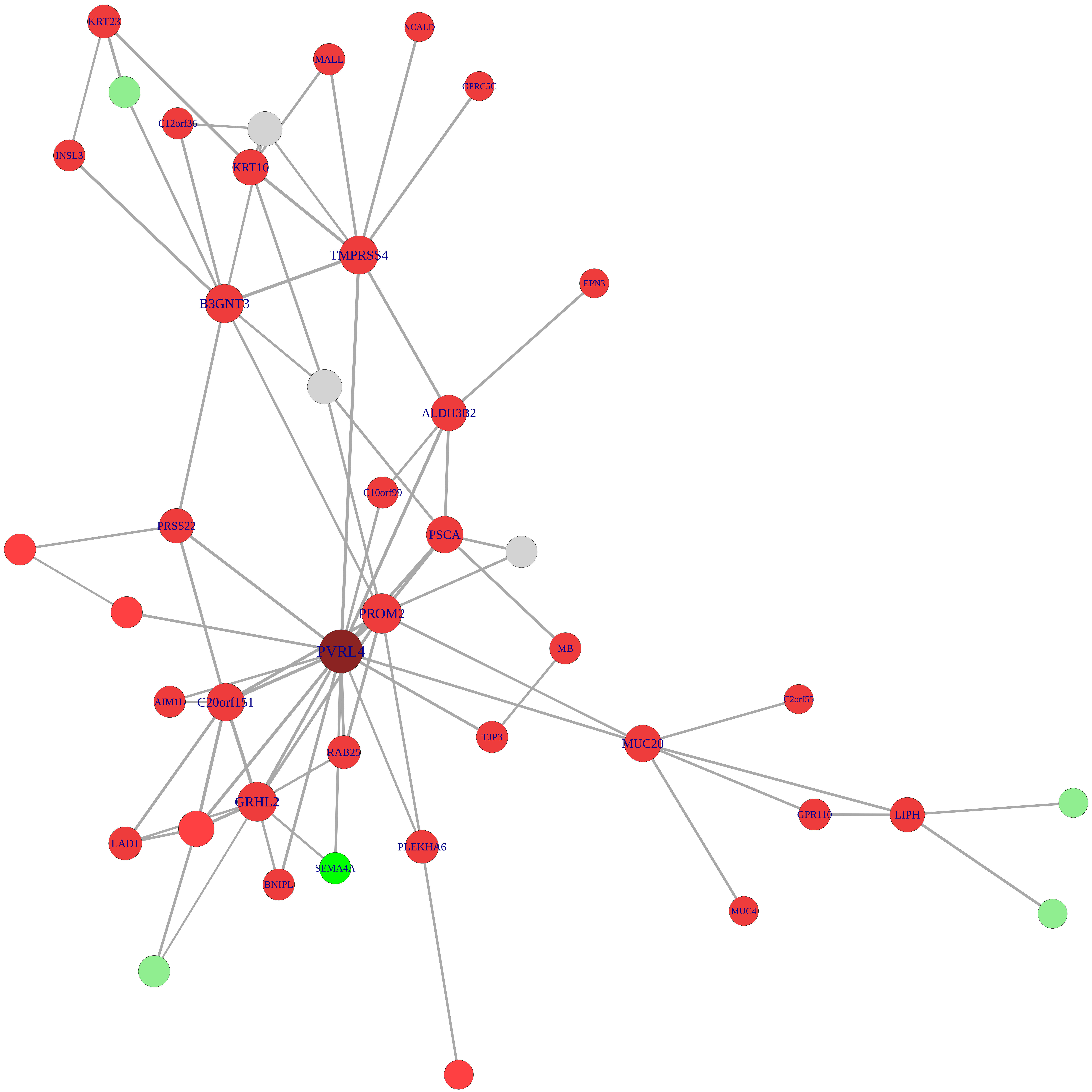
KRT23
NCALD
MALL
GPRC5C
C12orf36
INSL3
KRT16
TMPRSS4
EPN3
B3GNT3
ALDH3B2
C10orf99
PRSS22
PSCA
PROM2
MB
PVRL4
AIM1L
C20orf151
C2orf55
TJP3
MUC20
RAB25
GRHL2
GPR110
LIPH
LAD1
PLEKHA6
SEMA4A
BNIPL
MUC4
