# Supplementary material for: NECTIN4 (PVRL4) as Putative Therapeutic Target for a Specific Subtype of High Grade Serous Ovarian Cancer—An Integrative Multi-Omics Approach
Source: Cancers (Basel). 2019 May 20;11(5):698. doi: 10.3390/cancers11050698 (PMC6562934; doi:10.3390/cancers11050698)
Supplement: Supplementary file 1 [file c1_143_PM_Up__42_graph.pdf › cancers-491103-suppl-final/Tables S1 and S2/c1_143_PM_Up__42_graph.pdf]

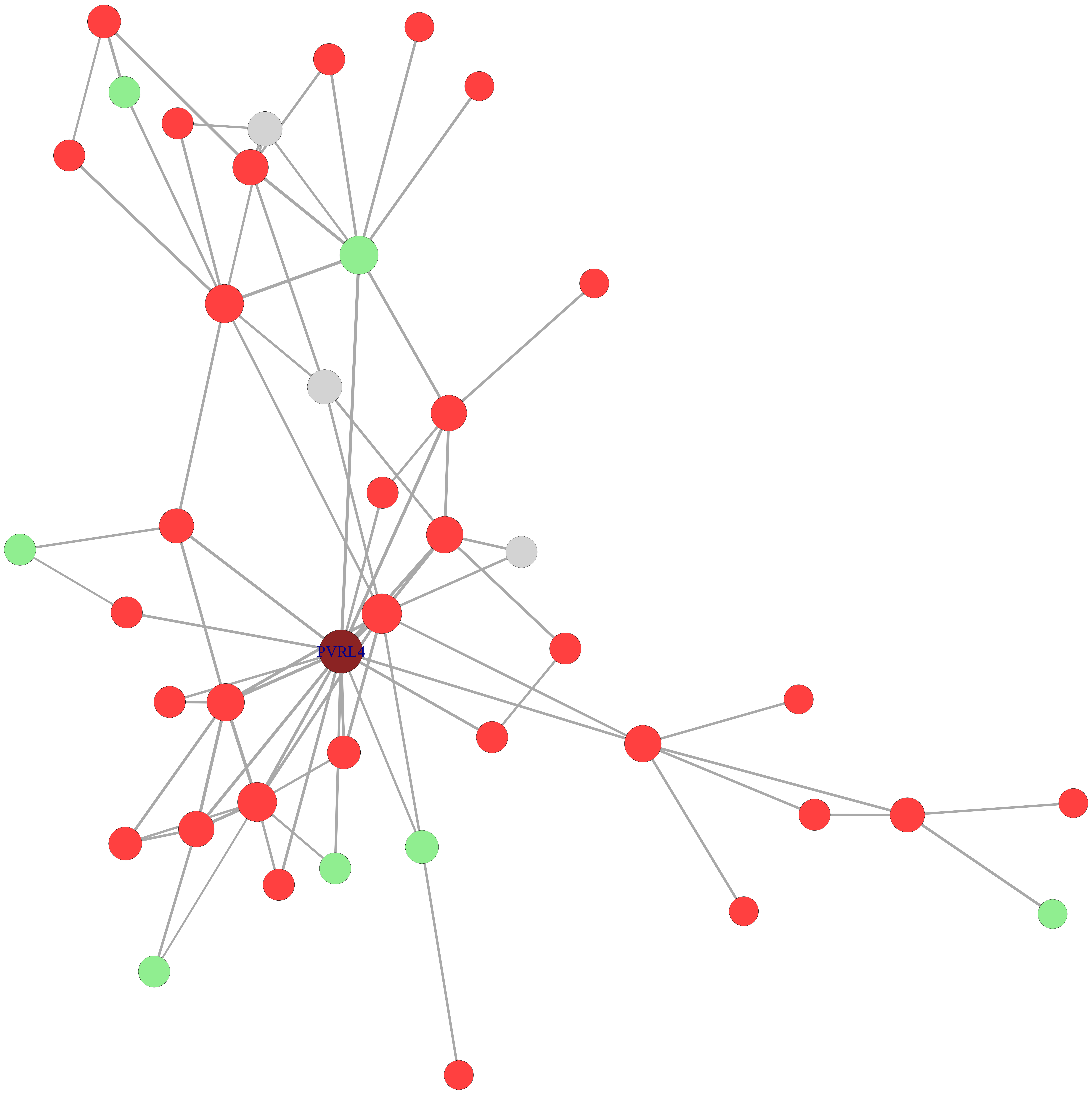
PVRL4
