# Supplementary material for: NECTIN4 (PVRL4) as Putative Therapeutic Target for a Specific Subtype of High Grade Serous Ovarian Cancer—An Integrative Multi-Omics Approach
Source: Cancers (Basel). 2019 May 20;11(5):698. doi: 10.3390/cancers11050698 (PMC6562934; doi:10.3390/cancers11050698)
Supplement: Supplementary file 1 [file c1_32_AS_Up__201_graph.pdf › cancers-491103-suppl-final/Tables S1 and S2/c1_32_AS_Up__201_graph.pdf]

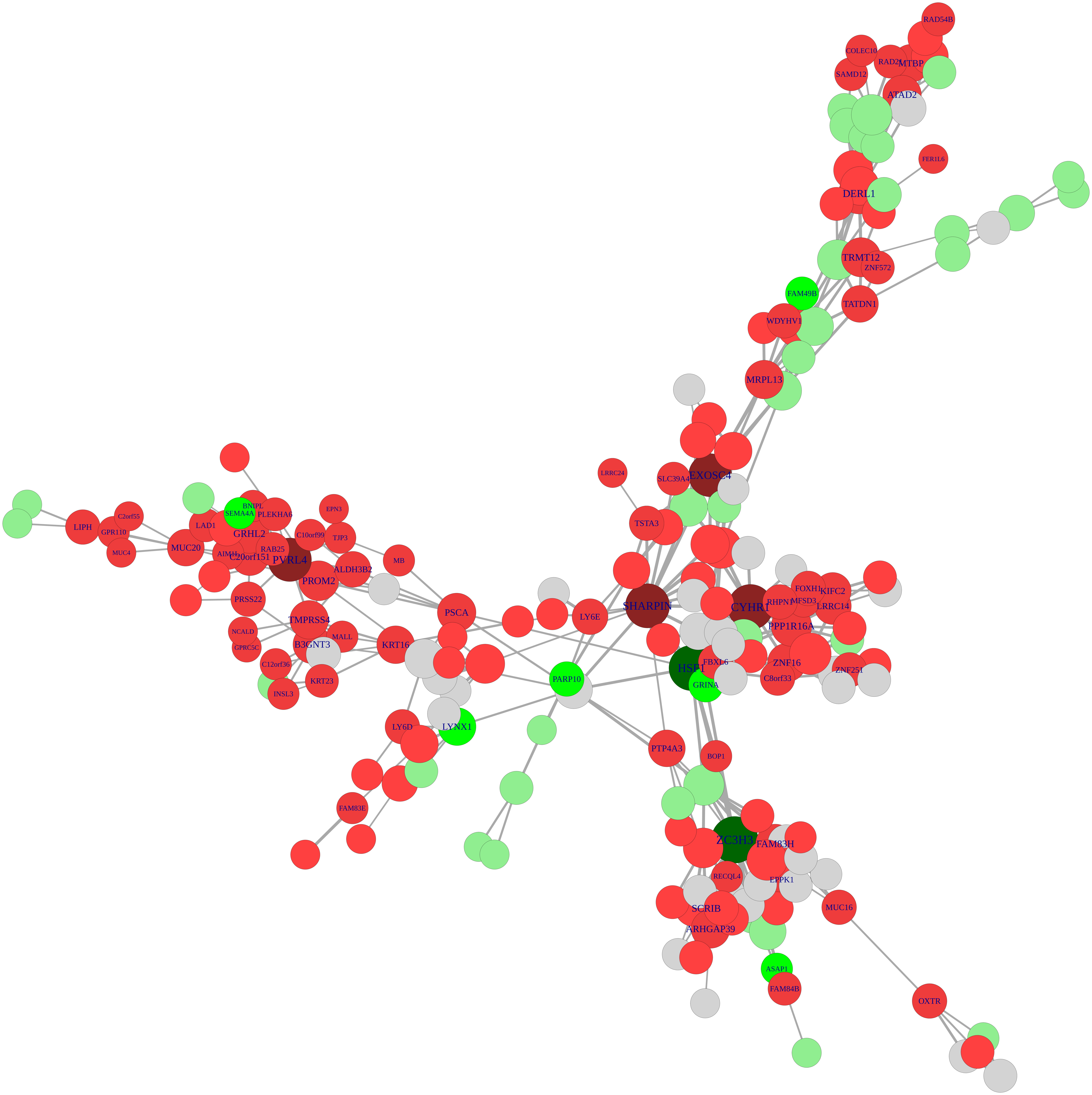

RAD54B
COLEC10
RAD21
MTBP
SAMD12
ATAD2
FER1L6
DERL1
TRMT12
ZNF572
FAM49B
TATDN1
WDYHV1
MRPL13
LRRC24
SLC39A4
EXOSC4
TSTA3
SHARPIN
CYHR1
RHPN1
FOXH1
MFSD3
KIFC2
LRRC14
PPP1R16A
ZNF16
ZNF251
C8orf33
FBXL6
HSF1
GRINA
LY6E
PARP10
PTP4A3
BOP1
ZC3H3
FAM83H
RECQL4
EPPK1
MUC16
SCRIB
ARHGAP39
ASAP1
FAM84B
OXTR
LIPH
C2orf55
GPR110
MUC4
MUC20
LAD1
BNIPL
SEMA4A
PLEKHA6
GRHL2
EPN3
C10orf99
TJP3
AIM1L
RAB25
C20orf151
PVRL4
ALDH3B2
MB
PROM2
PRSS22
PSCA
TMPRSS4
NCALD
GPRC5C
MALL
B3GNT3
KRT16
C12orf36
INSL3
KRT23
LY6D
LYNX1
FAM83E
